# Supplementary material for: Intimate partner violence victimisation in early adulthood: psychometric properties of a new measure and gender differences in the Avon Longitudinal Study of Parents and Children
Source: BMJ Open. 2019 Mar 23;9(3):e025621. doi: 10.1136/bmjopen-2018-025621 (PMC6475136; doi:10.1136/bmjopen-2018-025621)
Supplement: Supplementary data [file bmjopen-2018-025621supp001.pdf]

Table A1: Polychoric correlations between ordinal IPV items

	Control	Humiliate	Push, slap	Punch, strangle	Coerced touch	Forced touch	Coerced sex	Forced sex
**Control**	1							
**Humiliate**	.764	1						
**Push, slap**	.728	.812	1					
**Punch, strangle**	.727	.786	.923	1				
**Coerced touch**	.592	.570	.630	.589	1			
**Forced touch**	.591	.569	.677	.649	.923	1		
**Coerced sex**	.627	.598	.640	.591	.882	.827	1	
**Forced sex**	.593	.602	.742	.720	.812	.890	.854	1

Note. N=3,158. Response categories for all variables were 0=never, 1=once, 2=a few times, and 3=often. Full item descriptions are shown in Table 1.

Table A2: Exploratory factor analysis (total sample)

	**Single factor solution**		**Two-factor solution (r=64.97%)**		
**Item**	**Factor 1**	**Uniqueness**	**Factor 1**	**Factor 2**	**Uniqueness**
**Control**	.771	.406	-	.705	.344
**Humiliate**	.792	.373	-	.842	.249
**Push, slap**	.881	.224	-	.877	.106
**Punch, strangle**	.855	.269	-	.905	.127
**Coerced touch**	.870	.243	.951	-	.098
**Forced touch**	.891	.207	.913	-	.092
**Coerced sex**	.866	.251	.862	-	.156
**Forced sex**	.898	.193	.770	-	.147
**Factor eigenvalue:**	**5.834**		**5.834**	**0.847**	
**Proportion of variance explained**	**.865**		**.865**	**.126**	

Note. N=3,158. Method is principal factors using a polychoric correlation matrix. Two-factor solution uses oblique rotation (promax). Factor loadings <.4 are suppressed. Full item descriptions are shown in Table 1.

Table A3: Exploratory factor analysis (women only)

	**Single factor solution**		**Two-factor solution (r=63.86%)**		
**Item**	**Factor 1**	**Uniqueness**	**Factor 1**	**Factor 2**	**Uniqueness**
**Control**	.786	.382	-	.714	.322
**Humiliate**	.805	.351	-	.863	.220
**Push, slap**	.886	.215	-	.887	.096
**Punch, strangle**	.860	.260	-	.928	.106
**Coerced touch**	.858	.264	.948	-	.107
**Forced touch**	.878	.230	.929	-	.093
**Coerced sex**	.860	.261	.853	-	.162
**Forced sex**	.892	.205	.755	-	.158
**Factor eigenvalue:**	**5.832**		**5.832**	**0.903**	
**Proportion of variance explained**	**.853**		**.853**	**.132**	

Note. N=2,050 women. Method is principal factors using a polychoric correlation matrix. Two-factor solution uses oblique rotation (promax). Factor loadings <.4 are suppressed. Full item descriptions are shown in Table 1.

Table A4: Exploratory factor analysis (men only)

	Single factor solution		Two-factor solution (r=59.60%)		
**Item**	**Factor 1**	**Uniqueness**	**Factor 1**	**Factor 2**	**Uniqueness**
**Control**	.790	.377	-	.751	.294
**Humiliate**	.749	.439	-	.896	.248
**Push, slap**	.869	.245	-	.739	.188
**Punch, strangle**	.776	.398	-	.934	.189
**Coerced touch**	.876	.233	.928	-	.086
**Forced touch**	.926	.142	.646	-	.132
**Coerced sex**	.822	.325	.876	-	.192
**Forced sex**	.822	.325	.992	-	.104
**Factor eigenvalue:**	**5.518**		**5.518**	**1.049**	
**Proportion of variance explained**	**.690**		**.690**	**.131**	

Note. N=1,108 men. Method is principal factors using a polychoric correlation matrix, forced to be positive definite. Two-factor solution uses oblique rotation (promax). Factor loadings <.4 are suppressed. Full item descriptions are shown in Table 1.

Table A5: Polychoric correlations between IPV impact items

	Frightened	Upset	Affect work	Sad	Anxious	Depressed	Substance use	Angry	No effect	Loved	Funny
**Frightened**	1										
**Upset**	.717	1									
**Affect work**	.621	.721	1								
**Sad**	.663	.893	.799	1							
**Anxious**	.701	.693	.724	.631	1						
**Depressed**	.646	.732	.776	.778	.747	1					
**Substance use**	.379	.400	.480	.406	.452	.531	1				
**Angry**	.343	.660	.318	.558	.401	.426	.297	1			
**No effect**	-.627	-.862	-.590	-.820	-.686	-.678	-.264	-.631	1		
**Loved**	-.178	-.468	-.162	-.376	-.148	-.164	-.018	-.301	.419	1	
**Funny**	-.478	-.522	-.318	-.422	-.472	-.454	-.093	-.392	.639	.432	1

Note. N=1,092. Participants only responded if they had experienced any IPV victimisation. Response categories for all variables were 0=no, 1=yes. Full item descriptions are shown in Table 2.

Table A6: Age of occurrence among those who had experienced IPV

		N (%)		
**Item**	**Total N**	**Under 18**	**Over 18**	**Both**
Told you who you could see and where you could go and/or regularly checked what you were doing and where you were (by phone or text)	953	201 (21.09)	569 (59.71)	183 (19.20)
Made fun of you, called you hurtful names, shouted at you	1,022	171 (16.73)	666 (65.17)	185 (18.10)
Used physical force such as pushing, slapping, hitting or holding you down	743	137 (18.44)	468 (62.99)	138 (18.57)
Used more severe physical force such as punching, strangling, beating you up, hitting you with an object	481	70 (14.55)	293 (60.91)	118 (24.53)
Pressured you into kissing/touching/something else	569	109 (19.16)	337 (59.23)	123 (21.62)
Physically forced you into kissing/touching/something else	437	74 (16.93)	255 (58.35)	108 (24.71)
Pressured you into having sexual intercourse	631	135 (21.39)	371 (58.80)	125 (19.91)
Physically forced you into having sexual intercourse	413	65 (15.74)	244 (59.08)	104 (25.18)
