# Supplementary material for: Intimate partner violence victimisation in early adulthood: psychometric properties of a new measure and gender differences in the Avon Longitudinal Study of Parents and Children
Source: BMJ Open. 2019 Mar 23;9(3):e025621. doi: 10.1136/bmjopen-2018-025621 (PMC6475136; doi:10.1136/bmjopen-2018-025621)
Supplement: Author's manuscript [file bmjopen-2018-025621.draft_revisions.pdf]

BMJ Open

BMJ Open is committed to open peer review. As part of this commitment we make the peer review history of every article we publish publicly available.

When an article is published we post the peer reviewers' comments and the authors' responses online. We also post the versions of the paper that were used during peer review. These are the versions that the peer review comments apply to.

The versions of the paper that follow are the versions that were submitted during the peer review process. They are not the versions of record or the final published versions. They should not be cited or distributed as the published version of this manuscript.

BMJ Open is an open access journal and the full, final, typeset and author-corrected version of record of the manuscript is available on our site with no access controls, subscription charges or pay-per-view fees (http://bmjopen.bmj.com).

If you have any questions on BMJ Open's open peer review process please email editorial.bmjopen@bmj.com

BMJ Open

Intimate partner violence victimisation in early adulthood: psychometric properties of a new measure and gender differences in the Avon Longitudinal Study of Parents and Children

Journal:	*BMJ Open*
Manuscript ID	bmjopen-2018-025621
Article Type:	Research
Date Submitted by the Author:	27-Jul-2018

Complete List of Authors:	Yakubovich, Alexa; University of Oxford, Social Policy & Intervention<br>Feder, Gene; University of Bristol, Community based medicine<br>Heron, Jon; University of Bristol, School of Social and Community Medicine<br>Fraser, Abigail; University of Bristol, School of Social and Community Medicine<br>Humphreys, David; University of Oxford, Social Policy & Intervention

Keywords:	SOCIAL MEDICINE, PUBLIC HEALTH, MENTAL HEALTH, EPIDEMIOLOGY

SCHOLARONE™ Manuscripts

For peer review only

Intimate partner violence victimisation in early adulthood: psychometric properties of a new measure and gender differences in the Avon Longitudinal Study of Parents and Children

Alexa R. Yakubovich,[1] Jon Heron,[2] Gene Feder,[2] Abigail Fraser,[2] David K. Humphreys[1,3]

[1]Department of Social Policy and Intervention, University of Oxford, Bristol, United Kingdom
[2]School of Social and Community Medicine, University of Bristol, Bristol, United Kingdom
[3]Green Templeton College, University of Oxford, Oxford, United Kingdom

Correspondence to: Ms. Alexa R. Yakubovich, Department of Social Policy and Intervention, University of Oxford, Oxford, OX1 2ER, United Kingdom, alexa.yakubovich@spi.ox.ac.uk

Word count (excluding title page, abstract, references, and tables): 3,000 words

For peer review only

ABSTRACT

Objectives: To evaluate the psychometric properties of a novel, brief measure of physical, psychological, and sexual intimate partner violence (IPV) and estimate the overall prevalence of and gender differences in this violence.

Design: Data are from the Avon Longitudinal Study of Parents and Children (ALSPAC), a birth-cohort study.

Setting: Avon, United Kingdom.

Participants: 2,126 women and 1,142 men who completed the questionnaire assessment at age 21.

Outcome measures: Participants responded to eight items on physical, psychological, and sexual IPV victimisation at age 21. Participants indicated whether the violence occurred before age 18 and/or after and led to any of eight negative impacts (e.g., fear). We estimated the prevalence of IPV and tested for gender differences using chi-squared or t-tests. We evaluated the IPV victimisation measure based on internal consistency (alpha coefficient), dimensionality (exploratory factor analysis), and convergent validity with negative impacts.

Results: Overall, 37% of participants reported experiencing any IPV and 28% experienced any IPV after age 18. Women experienced more frequent IPV, more acts of IPV, and more negative impacts than men ($p<.001$ for all comparisons). The IPV measure showed high internal consistency ($\alpha=.95$), strong evidence for unidimensionality, and was highly correlated with negative impacts ($r=.579$, $p<.001$).

Conclusions: The prevalence of IPV victimisation in the ALSPAC cohort was considerable for both women and men. The strong and consistent gender differences in the frequency and severity of IPV suggests clinically meaningful differences in experiences of this violence. The ALSPAC measure for IPV victimisation was valid and reliable, indicating its suitability for further aetiological investigations.

Keywords: Epidemiology; Mental health; Public health; Social medicine

Strengths and limitations of this study:

- This study is the first to evaluate a novel and relatively brief measure of physical, psychological, and sexual intimate partner violence using data from a long running, high quality birth-cohort study in the United Kingdom.
- Timing of violence was measured which allowed us to compute both the lifetime and early adulthood prevalence of intimate partner violence.
- We used a robust analysis strategy to test for gender differences in intimate partner violence, which included analyzing the impacts of this violence to determine the severity of clinical burdens among women and men.
- Details on specific incidents or perpetrators of intimate partner violence were not measured.

For peer review only

INTRODUCTION

Intimate partner violence (IPV) is the most common violence perpetrated against women worldwide with severe consequences, including mortality, injury, and mental health disorders.[1-3] The most recent estimates for the United Kingdom (UK) indicate that IPV, especially among women, should be a public health priority, with 23% of women and 11% of men reporting any physical, psychological, or sexual IPV in their lifetime.[4 5] However, designing interventions for IPV requires accurately measuring and understanding its burden. Unlike many public health problems, official (e.g., police or hospital-reported) data typically provide poor estimates since most people do not contact formal services after experiencing IPV.[6] One meta-synthesis of qualitative studies found that barriers to disclosure to healthcare professionals included women's feelings regarding the abuse (e.g., shame), fear of the repercussions, and perceptions of the professionals (e.g., as not respecting confidentiality).[7] These factors result in an inadequate healthcare response to and further prevention of IPV against women as well as its underestimation in official data.

Although survey data on IPV tend to be viewed as more accurate, measurement quality varies widely. While single-term and vague items such as *violence* are insufficient to measure the complexity of IPV, multi-item scales vary in content and length. The most commonly used measure is currently the Conflict Tactics Scale,[8 9] which measures specific behaviours by a current or previous dating, cohabiting, or marital partner. However, the Conflict Tactics Scale has been criticised for measuring IPV only within the context of conflicts, disagreements, or 'settling differences' and not measuring the intent (e.g., self-defense or harm) or impact of violence.[10 11] Other validated scales include the Composite Abuse Scale,[12] WHO multi-country survey,[13] Abusive Behavior Inventory,[14] Severity of Violence Against Women Scale,[15] and Measure of Wife Abuse.[16] However, several of these do not measure psychological IPV[13 15] and most are relatively long (>30 items), risking response burden in larger or repeated-measures surveys.

We aimed to (1) evaluate the psychometric properties of a new instrument for measuring IPV; (2) estimate the overall prevalence of IPV and its impacts; and (3) test for gender differences in a UK-based birth-cohort study. This is essential to developing aetiological evidence for IPV against women, which, as demonstrated by a recent systematic review of prospective-longitudinal studies, is severely limited outside the United States (US): Only 12 of 60 studies investigating any risk or protective factor for IPV against women were based outside the US, one of which was from the UK.[17]

METHOD

This study uses data from the Avon Longitudinal Study of Parents and Children (ALSPAC), which provides a unique opportunity to evaluate a new, brief measure of physical, psychological, and sexual IPV. The birth-cohort study has established trust among participants, who have been self-completing questionnaires since age 5 (now in early adulthood), using online questionnaires at later time points – both of which are ideal for measuring IPV. ALSPAC also measured specific IPV experiences and their impacts, without referencing any context like conflict resolution. The sampling frame included all pregnant women resident in one of three health districts in Avon, UK with an expected due date between 1 April 1991 and 31 December 1992.[18 19] The initial number of pregnancies enrolled was 14,541. When participating children were approximately age 7, eligible cases not in the study were contacted. The sample size resultantly increased to 15,427 pregnancies, with

For peer review only

14,775 live births (76% of eligible live births) – these children are our target sample. Ethical approval was obtained from the ALSPAC Ethics and Law Committee and Local Research Ethics Committees. Details are available through a fully searchable data dictionary at http://www.bristol.ac.uk/alspac/researchers/our-data/.

Measuring IPV

At age 21, 3,458 participants completed the online questionnaire, of whom 3,268 (2,126 women, 1,142 men) provided any data on IPV, making this our starting sample. The IPV measures described below (see also Table 1) were based on a previous National Society for Prevention of Cruelty to Children questionnaire used in a young population in Bristol,[20] with modified wording based on the PROVIDE questionnaire.[21] The development group consisted of IPV researchers (Christine Barter, Marianne Hester, Eszter Szilassy, and Gene Feder); the questionnaire was piloted for acceptability with the ALSPAC participant advisory group.

Main instrument: IPV victimisation

Eight items measured physical, psychological, and sexual IPV victimisation. A ninth item in this scale (feeling scared) was relevant to the impact of this violence and therefore is included with the impact items. Participants indicated whether each item occurred before and/or after age 18, allowing for measurement of temporality.

Impacts of IPV

Ten items measured the psychological impacts of IPV. Eight items indicated negative impacts (e.g., upset). One item measured whether the violence had no effect and two measured whether it led to positive effects (e.g., feeling loved).

Table 1: IPV victimisation and impact items

Order	Victimisation items: How often altogether have any of your partners ever done any of the following to you and how old were you?	Type of IPV
1	Told you who you could see and where you could go and/or regularly checked what you were doing and where you were (by phone or text)?	Psychological
2	Made fun of you, called your hurtful names, shouted at you?	Psychological
3	Used physical force such as pushing, slapping, hitting or holding you down?	Physical
4	Used more severe physical force such as punching, strangling, beating you up, hitting you with an object?	Physical
5	Pressured you into kissing/touching/something else?	Sexual
6	Physically forced you into kissing/touching/something else?	Sexual
7	Pressured you into having sexual intercourse?	Sexual
8	Physically forced you into having sexual intercourse?	Sexual
**Order**	**Impact items: How did you feel after they these things to you?**	**Dimension**
1	Did any of the above make you feel scared or frightened, or did any partner make you feel frightened in any other way?*	Negative
2	Upset/unhappy	Negative
3	Affected my work/studies	Negative
4	Made me feel sad	Negative
5	No effect/not bothered	Null
6	Anxious	Negative
7	Made me drink more alcohol/take more drugs	Negative
8	Felt loved/protected/wanted	Positive
9	Thought it was funny	Positive
10	Angry/annoyed	Negative
11	Depressed	Negative

Note. For each victimisation item, participants indicate the frequency of occurrence – where 0=never, 1=once, 2=a few times, 3=often – and age of occurrence, where 1=under 18, 2=over 18, 3=both. The question prompt included the following definition for partner: 'By partner we mean anyone you have ever been out with or had a relationship with, long-term or short-term (including one night stands)'. For each impact item, participants indicated 'yes' or 'no' as to whether this is how the IPV they experienced affected them.
*This item was asked along with the victimisation items and was therefore measured on the 'frequency' response scale.

For peer review only - http://bmjopen.bmj.com/site/about/guidelines.xhtml

Analysis

Analyses were run in Stata v.13.1 and RStudio v.1.0.136. For aim one, we evaluated the internal consistency, dimensionality, and convergent validity of the IPV victimisation scale. To determine internal consistency, we computed an alpha coefficient for the eight IPV victimisation items using the polychoric (rather than Pearson) correlation matrix, which accounts for variables being ordinal rather than continuous and provides more reliable coefficients even when the underlying latent variables are not normally distributed as assumed.[22] To determine the scale's dimensionality, we conducted an exploratory factor analysis using this correlation matrix.[23] We decided the appropriate number of factors based on their eigenvalues (using Kaiser's criterion that >1 indicates a viable factor), scree plot, and theoretical plausibility.[24] If a two (or more) factor solution was favourable, we decided a priori to use oblique rotation since we expected differing dimensions of abuse to correlate. To establish convergent validity, we computed the Pearson correlation between the average frequency of IPV experiences and sum total of negative impacts among who those who had experienced any IPV. For this step, we first confirmed (via polychoric correlation) that the negative impacts of IPV were positively correlated with each other and negatively correlated with the positive and null impacts (categorisations are in Table 1).

For aim two, we computed the prevalence and frequency of IPV experiences and impacts. For aim three, we tested for gender differences using (a) chi-squared tests, to compare the proportions of women and men who reported that each IPV item had occurred at least once and the different impacts of this violence, and (b) two-sided t-tests, to compare women's and men's scores on the overall scale. When there was strong evidence to suggest that the variances of women's and men's scores were unequal (using the Levene's test), we computed a two-sided t-test for unequal variances.

RESULTS

Table 2 summarises sample characteristics by gender. Women and men were very similar on baseline socio-demographics: most were White and had characteristics of higher socioeconomic status. At age 21, most women and men saw themselves as completely heterosexual (83% women, 85% men), followed by a smaller proportion reporting at least some same-sex preferences (16% women, 13% men) and a small number indicating asexuality (<1%). More women (72%) than men (59%), however, had been in relationships longer than 3 months by age 18 and, by age 20, more women (12%) than men (6%) were living with partners or children.

Table 2: Socio-demographic characteristics of the sample by gender

	Women	Men
**Baseline**		
Ethnicity		
Non-White	134 (3.64)	138 (3.74)
White	3,545 (96.36)	3,552 (96.26)
At least one parent had higher than O-level education		
Yes	3,224 (55.29)	3,400 (54.76)
No	2,607 (44.71)	2,809 (45.24)
At least one parent part of lower social class (partly or unskilled occupation)		
Yes	1,150 (23.76)	1,167 (22.87)
No (Both parents in professional, managerial, or skilled occupations)	3,690 (76.24)	3,936 (77.13)
Mother married		
Yes	4,807 (75.30)	5,100 (74.53)
No	1,577 (24.70)	1,743 (25.47)

For peer review only - http://bmjopen.bmj.com/site/about/guidelines.xhtml

	Women	Men
Lived with both biological parents		
Yes	4,489 (90.29)	4,830 (90.26)
No	483 (9.71)	521 (9.74)
**Early adulthood (Ages 18-21)**		
Longest relationship (at Age 18)		
More than 3 months	1,632 (72.18)	1,034 (58.78)
Less than or equal to 3 months	629 (27.82)	725 (42.22)
Living arrangements (at Age 20):		
One or both parents	1,200 (48.21)	819 (51.51)
Partner and/or children	307 (12.33)	98 (6.16)
Other	982 (39.45)	673 (42.33)
Sexual preference (at Age 21)		
Asexual	8 (0.37)	6 (0.51)
Any same-sex preferences	358 (16.63)	160 (13.72)
100% heterosexual	1,787 (83.00)	1,000 (85.76)

Reliability and validity

Correlations were strong between all IPV scale items, ranging from .57 (between experiencing humiliation/name-calling/shouting and forced sexual touch) to .92 (between forced and coerced touch) (see Appendix, Table A1 for matrix). The alpha coefficient was .95, indicating strong internal consistency.

The exploratory factor analysis suggested a one- or two-factor solution (see Appendix, Table A2 for factor loadings). Only the first factor had an eigenvalue more than 1 (5.834). All items loaded highly onto this factor (ranging from 0.771-0.898), which suggests that using a factor-based score for experiences of IPV overall would be a valid analytical method in this sample. The scree plot plateaued between the second and third factor, and as the second factor had an eigenvalue close to 1 (0.847), we also attempted a two-factor solution with oblique rotation. This two-factor solution fit the data well, indicating plausible dimensions for (a) physical and psychological IPV and (b) sexual IPV. This suggests that analyses using a latent variable approach could reliably analyse these two factors. The factor analysis did not support a three-factor solution: the third factor had a low eigenvalue (0.182) and no items with a loading greater than .30.

As expected, the eight negative impact items were all positively correlated (ρ=.297 to .893, see Appendix, Table A3). These items were also negatively correlated with IPV having no impact (ρ=-.264 to -.862) or IPV seeming funny or increasing perceptions of being loved, protected, or wanted (ρ=-.018 to -.522). Finally, these three null or positive impacts were positively correlated (ρ=.419 to .639). We therefore, as planned, correlated the sum total of the negative impacts of IPV (minimum=0, maximum=8) with the average frequency of IPV experiences (maximum=3) among those who had experienced any IPV. As expected, experiencing more frequent IPV was strongly correlated with experiencing more negative impacts (N=1,111): r=.579, p<.001.

Overall prevalence

Table 3 summarises the frequencies of IPV experiences and impacts. The most frequently experienced IPV was psychological (e.g., 25% reported humiliation, name-calling, or shouting) and the least experienced was sexual (e.g., 4% reported forced sex). Among those who experienced any IPV, the majority of violent acts (>78%) occurred after age 18 (see Table A4, Appendix for more detail). Most participants reported at least one negative impact following IPV, with the most common being feeling upset (78%) or angry (75%). The least

common impacts of IPV were the positive ones: 13% of participants reported that the violence made them feel loved, protected, or wanted and 14% found the violence amusing.

Overall, 37% of participants reported experiencing any IPV and 28% experienced any IPV after age 18. The mean number of IPV acts experienced among those who experienced any violence, ranging from 1 to 8, was 3.004 (SD=2.108) overall and 2.167 (SD=1.644) after age 18. The mean number of negative impacts, ranging from 0 to 8, was 3.950 (SD=2.371) among those who had experienced any IPV and 2.944 (SD=2.633) among those who had experienced IPV after age 18.

For peer review only

Table 3: Frequencies of 8 IPV victimisation and impact items

Victimisation items	Total N	N (%) Never	Once	A few times	Often
Told you who you could see and where you could go and/or regularly checked what you were doing and where you were (by phone or text)	3,268	2,544 (77.85)	124 (3.79)	322 (12.91)	178 (5.45)
Made fun of you, called you hurtful names, shouted at you	3,253	2,422 (74.45)	170 (5.23)	530 (16.29)	131 (4.03)
Used physical force such as pushing, slapping, hitting or holding you down	3,255	2,768 (85.04)	193 (5.93)	235 (7.22)	59 (1.81)
Used more severe physical force such as punching, strangling, beating you up, hitting you with an object	3,252	3,075 (94.56)	81 (2.49)	68 (2.09)	28 (0.86)
Pressured you into kissing/touching/something else	3,255	2,981 (96.58)	96 (2.95)	146 (4.49)	32 (0.98)
Physically forced you into kissing/touching/something else	3,250	3,115 (95.85)	68 (2.09)	49 (1.51)	18 (0.55)
Pressured you into having sexual intercourse	3,242	2,876 (88.71)	181 (5.58)	152 (4.69)	33 (1.02)
Physically forced you into having sexual intercourse	3,239	3,118 (96.26)	80 (2.47)	32 (0.99)	9 (0.28)
**Impact items**	**Total N**	**Never**	**Once**	**A few times**	**Often**
Scared or frightened in any way	3,221	2,711 (84.17)	191 (5.93)	234 (7.26)	85 (2.64)
**Impact items: Only those who experienced at least 1 act of IPV**	**Total N**	**Yes**		**No**	
Upset/unhappy	1,148	900 (78.40)		248 (21.60)	
Angry/annoyed	1,139	857 (75.24)		282 (24.76)	
Made me feel sad	1,142	813 (71.19)		329 (28.81)	
Affected my work/studies	1,141	799 (70.03)		342 (29.97)	
Anxious	1,133	495 (43.69)		638 (56.31)	
Depressed	1,138	418 (36.73)		720 (63.27)	
No effect/not bothered	1,133	206 (18.18)		927 (81.82)	
Made me drink more alcohol/take more drugs	1,138	168 (14.76)		970 (85.24)	
Thought it was funny	1,132	158 (13.96)		974 (86.04)	
Felt loved/protected/wanted	1,135	148 (13.04)		987 (86.96)	

For peer review only

Gender differences

As shown in Table 4, for all IPV victimisation items, regardless of whether lifetime or early adulthood (ages 18-21) was considered, significantly more women experienced violence than men. The largest percentage difference was for the lifetime prevalence of coerced sex (15% women, 4% men). Moreover, significantly more women than men reported experiencing all negative impacts of IPV, apart from substance use where there was no difference. The greatest percentage difference was in feeling scared because of their partner (56% women, 14% men in their lifetime). In contrast, more men than women reported that the IPV they experienced was funny or had no effect on them. Finally, every test indicated that women experienced more frequent and severe IPV overall than men, in both their lifetimes and early adulthood (see Table 5): women experienced more frequent and more acts of IPV compared to men; more women than men experienced any IPV (with or without a negative impact); and, among those who had experienced any IPV, women experienced more negative impacts than men.

For peer review only

Table 4: Gender differences in IPV victimisation and impact items

Victimisation items	Lifetime Women (N=2,050)	Men (N=1,108)	χ^2 (p)	Ages 18-21 Women (N=2,014)	Men (N=1,092)	χ^2 (p)
Told you who you could see, where you could go, or regularly checked what you were doing and where you were	510 (24.88)	196 (17.69)	21.41 (<.001)	346 (17.18)	152 (13.92)	5.59 (.018)
Made fun of you, called you hurtful names, shouted at you	596 (29.07)	210 (18.95)	38.75 (<.001)	443 (22.00)	166 (15.20)	20.74 (<.001)
Used physical force such as pushing, slapping, hitting or holding you down	362 (17.66)	106 (9.57)	37.31 (<.001)	245 (12.16)	82 (7.51)	16.29 (<.001)
Used more severe physical force such as punching, strangling, beating you up, hitting you with an object	142 (6.93)	31 (2.80)	23.68 (<.001)	96 (4.77)	22 (2.01)	14.67 (<.001)
Pressured you into kissing/touching/something else	240 (11.71)	26 (2.35)	81.70 (<.001)	144 (7.15)	20 (1.83)	40.05 (<.001)
Physically forced you into kissing/touching/something else	125 (6.10)	7 (0.63)	53.65 (<.001)	72 (3.57)	5 (0.46)	28.46 (<.001)
Pressured you into having sexual intercourse	313 (15.27)	43 (3.88)	93.25 (<.001)	192 (9.53)	36 (3.30)	40.49 (<.001)
Physically forced you into having sexual intercourse	114 (5.56)	5 (0.45)	51.79 (<.001)	64 (3.18)	5 (0.46)	24.12 (<.001)
**Impact items (Among those who experienced any IPV)**	**Women (N=800)**	**Men (N=292)**	**χ^2 (p)**	**Women (N=552)**	**Men (N=221)**	**χ^2 (p)**
Scared	444 (55.50)	40 (13.70)	151.47 (<.001)	279 (50.54)	31 (14.03)	87.61 (<.001)
Upset/unhappy	684 (85.50)	179 (61.30)	75.58 (<.001)	465 (84.24)	141 (63.80)	38.92 (<.001)
Angry/annoyed	625 (78.12)	195 (66.78)	14.72 (<.001)	441 (79.89)	152 (68.78)	10.91 (.001)
Made me feel sad	621 (77.62)	157 (53.77)	59.44 (<.001)	425 (76.99)	122 (55.20)	36.22 (<.001)
Affected my work/studies	275 (34.38)	51 (17.47)	29.21 (<.001)	187 (33.88)	38 (17.19)	21.28 (<.001)
Anxious	406 (50.75)	73 (25.00)	57.60 (<.001)	272 (49.28)	60 (27.15)	31.53 (<.001)
Depressed	329 (41.12)	69 (23.63)	28.27 (<.001)	231 (41.85)	52 (23.53)	22.82 (<.001)
No effect/not bothered	109 (13.63)	89 (30.48)	40.94 (<.001)	74 (13.41)	59 (26.70)	19.57 (<.001)
Made me drink more alcohol/take more drugs	127 (15.88)	34 (11.64)	3.05 (.081)	92 (16.67)	30 (13.57)	1.14 (.287)
Thought it was funny	64 (8.00)	92 (31.51)	96.53 (<.001)	48 (8.70)	67 (30.32)	58.26 (<.001)
Felt loved/protected/wanted	101 (12.62)	41 (14.04)	0.38 (.538)	80 (14.49)	32 (14.48)	0.00 (.996)

Note. Victimisation items were coded as 1=experienced at least once, 0=never experienced. Impact items were 1=yes, 0=no.

Table 5: Summary statistics for comparisons between women and men on overall IPV victimisation and impact

	Lifetime						Ages 18-21					
	Women		Men				Women		Men			
Item	N	M (SD) or N (%)	N	M (SD) or N (%)	t(df) or χ^2	p	N	M (SD) or N (%)	N	M (SD) or N (%)	t(df) or χ^2	p
Mean frequency of IPV experiences (SD)	2,128	0.28 (0.50)	1,145	0.12 (0.25)	12.61 (3,252.18)	<.001	2,128	0.19 (0.39)	1,145	0.10 (0.24)	7.58 (3,219.11)	<.001
Mean number of IPV acts experienced (SD)	2,024	1.41 (2.19)	1,096	0.60 (1.22)	13.16 (3,115.22)	<.001	2,014	0.75 (1.47)	1,092	0.42 (0.97)	7.55 (2,996.44)	<.001
Any IPV (N, %)	2,024	851 (42.05)	1,096	318 (29.01)	51.53	<.001	2,014	612 (30.39)	1,092	250 (22.89)	19.83	<.001
Any IPV with a negative impact (N, %)	1,988	762 (38.33)	1,071	228 (21.29)	92.34	<.001	1,982	535 (26.99)	1,070	180 (16.82)	40.07	<.001
Mean number of negative impacts of IPV (SD)	800	4.39 (2.27)	292	2.73 (2.21)	10.75 (1,090)	<.001	746	3.21 (2.72)	279	2.24 (2.24)	5.77 (602.84)	<.001

Note. All t-tests were two-group t-tests with unequal variances, apart from 'number of negative impacts of IPV' for the overall sample, which did not have unequal variances between men and women (i.e., the Levene's test was statistically non-significant).

For peer review only - http://bmjopen.bmj.com/site/about/guidelines.xhtml

DISCUSSION

This study estimated the prevalence of physical, psychological, and sexual IPV in a UK birth cohort during early adulthood using a novel measure. The prevalence of IPV was high: 37% of participants had experienced any IPV in their lifetime and 28% had experienced IPV between ages 18 to 21. As in previous research, the most commonly experienced violence was psychological and the least commonly experienced was sexual.[5] Over three-quarters of those who had experienced IPV had experienced this violence when they were aged 18 or older. This aligns with the broader IPV literature, which has found that early adulthood is an especially high-risk period for experiencing IPV.[17] Most participants who had experienced IPV reported more than one negative psychological impact, with the most common being feeling upset or angry. The least common outcomes of IPV were finding the violence amusing or feeling more loved, wanted, or protected.

We found strong and consistent gender differences: for all types of violent behaviours, women experienced more frequent IPV than men, both in their lifetime and early adulthood. As in other prevalence surveys, the most dramatic differences between women and men were on sexual violence items.[5] For instance, the proportion of women who had ever experienced coerced sex was more than four times that of men. Moreover, significantly more women than men reported experiencing negative psychological impacts from IPV. For example, the proportion of women who felt afraid of their partner was more than four times that of men. Similar proportions of women and men reported that their alcohol and substance use increased after experiencing IPV. The evidence on whether there are gender differences in substance use following IPV is inconsistent;[25] one possible explanation for similar proportions is the greater psychological impacts of IPV among women balance with the greater baseline tendency among men to use substances.

In contrast, the proportion of men who found their experiences of IPV amusing was more than three times that of women. More than double the proportion of men also reported that this violence did not affect them. Together with the gender differences in the negative impacts of IPV, this suggests that women experience more severe IPV than men, which is more difficult to trivialise and more likely to cause psychological harm. This extends a large body of evidence demonstrating that women experience the majority of severe consequences of IPV and are more likely to have controlling, violent partners.[2 26] At the same time, participants' reporting on the impacts and interpretations of their IPV experiences may have been influenced by gender-role socialisation. Women may have more readily reported the negative consequences of violence from their partners whereas men may have felt more pressure to minimise their experiences and deny negative consequences due to internalised concepts of masculinity, for instance, as strong and powerful and femininity as vulnerable and weak.[27-29] Future survey research could explore this hypothesis by including questions on traditional gender-role attitudes.

Our findings contribute to the broader debate on gender asymmetry in IPV. Studies using the Conflict Tactics Scale or family conflict surveys tend to find equivalent prevalence estimates among women and men;[30] whereas crime or clinical surveys tend to find that women experience more IPV than men.[11 30] Our data came from a community-based birth-cohort study, using a measure without reference to crime or conflict resolution, which thus minimises these priming biases. Our findings demonstrate that women experience more frequent and severe IPV than men, but also confirm that a considerable number of men experience violence from their partners. Therefore, our results support the continued research

For peer review only

and advocacy enterprise for IPV against women in particular, while also demonstrating the need for resources to continue to be developed for both women and men.

The new measure for IPV tested in this study showed excellent internal consistency and a strong, positive correlation with negative impacts of IPV, indicating convergent validity. The exploratory factor analysis suggested that the measure could be reliably analysed as a single dimension of IPV or as two – (a) physical or psychological IPV and (b) sexual IPV. As the scale items all loaded highly onto a single factor, analysing a factor-based score would be appropriate and have the benefit of maintaining the measure's original scaling for more intuitive interpretation.[31]

Strengths and limitations

Study limitations include, firstly, not measuring the age of first occurrence of IPV for those who experienced this violence prior to age 18. Second, the definition of intimate partner used was broad, from casual sex partners to long-term relationships. Since this could capture sexual violence by an acquaintance, it would be instructive for future research to use a more constrained definition (e.g., dating or marital partner). Third, the ALSPAC instrument did not measure specific instances of IPV or specific relationships; it is therefore unclear whether IPV was experienced by multiple perpetrators or repeatedly during a single relationship. We are also unable to determine which types or instances of IPV caused the impacts reported. It would be useful if future uses of the ALSPAC instrument allowed participants to indicate the perpetrator(s) and impact(s) caused by each experience of IPV. Obtaining more detailed information on IPV events would also be beneficial for determining intent and precipitants: although more time intensive, this could help inform directions for intervention research.

Fourth, the IPV impacts measured were mainly psychological, to the exclusion of further physical (e.g., injury) or socioeconomic consequences (apart from work or studies being affected), which contribute to the negative health burden of IPV.[32] Additionally, the impact items were largely one-word terms such as *depressed* and *anxious*, which are more vulnerable to social desirability bias and internalised gender concepts as opposed to a scale of items measuring depression or anxiety.[33] Nevertheless, including more exhaustive measures of IPV impacts may not be feasible in longer surveys, such as those used in ALSPAC.

Despite these limitations, our study has a number of strengths. The IPV measure used was brief and could therefore be implemented in surveys that measure a rich set of potential IPV predictors. The items were gender neutral and can therefore measure IPV among women and men. Although age of first occurrence was not measured, participants indicated whether IPV occurred before and/or after age 18, which made it possible to compute a current prevalence of IPV (last three years) and allows for analyses of temporal relationships between antecedents and early adulthood IPV. Finally, our analyses were thorough and demonstrated consistent results, allowing for firm conclusions on the reliability of the IPV measure and gender differences in IPV in the ALSPAC cohort.

In conclusion, we found that more than a third of participants engaged in a cohort study for over 20 years in the UK had experienced IPV by early adulthood. Women consistently experienced more frequent and severe IPV than men by all measures, suggesting important gender differences in the burden of this violence. The ALSPAC measure for IPV victimisation showed strong indicators of reliability and validity, demonstrating its appropriateness for further etiological studies.

For peer review only

Funding

The UK Medical Research Council and Wellcome (Grant ref: 102215/2/13/2) and the University of Bristol provide core support for ALSPAC. A comprehensive list of grants funding is available on the ALSPAC website (http://www.bristol.ac.uk/alspac/external/documents/grant-acknowledgements.pdf); this research was specifically funded by NHS Bristol Clinical Commissioning Group (RP-PG-0108-10048). ARY is funded by The Rhodes Trust and the Canadian Institutes of Health Research (DFS152265). AF and JH work at the MRC Integrative Epidemiology Unit which receives infrastructure funding from the UK Medical Research Council (MRC) (MC_UU_12013). AF is funded by a UK MRC fellowship awarded to AF (MR/M009351/1). GF is supported by the NIHR Biomedical Research Centre at University Hospitals Bristol NHS Foundation Trust and the University of Bristol.

Acknowledgments

The authors would like to thank all participants of ALSPAC, the midwives who helped recruit them, and the whole ALSPAC team, including the interviewers, computer and laboratory technicians, clerical workers, research scientists, volunteers, managers, receptionists, and nurses.

Author contributions

ARY and DKH designed this study. ARY conducted the analyses, interpreted the data, and drafted the manuscript. GF, JH, AF, and DKH provided comments on the manuscript. All authors have read and approved this final version. This manuscript is the work of the authors, who will serve as guarantors for its contents. The views expressed in this manuscript are those of the author(s) and not necessarily those of the NHS, the National Institute for Health Research, or the Department of Health.

Competing interests None.

References

1. Krug EG, Dahlberg LL, Mercy JA, et al., editors. *World report on violence and health*. Geneva: World Health Organization, 2002.
2. Stöckl H, Devries K, Rotstein A, et al. The global prevalence of intimate partner homicide: a systematic review. *The Lancet* 2013;382(9895):859-65. doi: 10.1016/s0140-6736(13)61030-2
3. Coker AL, Davis KE, Arias I, et al. Physical and mental health effects of intimate partner violence for men and women. *Am J Prev Med* 2002;23(4):260-68. doi: 10.1016/s0749-3797(02)00514-7
4. Flatley J. Crime in England and Wales QMI. UK2018.
5. Flatley J. Domestic abuse findings from the Crime Survey for England and Wales year ending March 2017. UK: Office for National Statistics 2018.
6. Flatley J. Intimate personal violence and partner abuse. UK: Office for National Statistics 2016.
7. Feder GS, Hutson M, Ramsay J, et al. Women exposed to intimate partner violence - Expectations and experiences when they encounter health care professionals: A meta-analysis of qualitative studies. *Archives of Internal Medicine* 2006;166:22-37.

8. Straus MA. Measuring intrafamily conflict and violence: The conflict tactics (CT) scales. *Journal of Marriage and the Family* 1979:75-88.
9. Straus MA, Hamby SL, Boney-McCoy S, et al. The revised conflict tactics scales (CTS2) development and preliminary psychometric data. *Journal of family issues* 1996;17(3):283-316.
10. Walby S, Towers J, Balderston S, et al. The Concept and Measurement of Violence Against Women and Men. Bristol: Policy Press 2017.
11. Kimmel MS. "Gender symmetry" in domestic violence: A substantive and methodological research review. *Violence Against Women* 2002;8(11):1332-63. doi: 10.1177/107780102237407
12. Hegarty K, Bush R, Sheehan M. The Composite Abuse Scale: Further development and assessment of reliability and validity of a multidimensional partner abuse measure in clinical settings. *Violence and Victims* 2005;20(5):529-47.
13. García-Moreno C, Jansen HA, Ellsberg M, et al., editors. *WHO multi-country study on women's health and domestic violence against women: Initial results on prevalence, health outcomes and women's responses*. Geneva: World Health Organization, 2005.
14. Shepard MF, Campbell JA. The Abusive Behavior Inventory: A measure of psychological and physical abuse. *Journal of interpersonal violence* 1992;7(3):291-305.
15. Marshall LL. Development of the Severity of Violence Against Women Scales. *Journal of Family Violence* 1992;7(2):103-21.
16. Rodenburg FA, Fantuzzo JW. The Measure of Wife Abuse: Steps toward the development of a comprehensive assessment technique. *Journal of Family Violence* 1993;8(3):203-28.
17. Yakubovich AR, Stöckl H, Murray J, et al. Risk and Protective Factors for Intimate Partner Violence Against Women: Systematic Review and Meta-analyses of Prospective–Longitudinal Studies. *American Journal of Public Health* 2018;108(7):e1-e11. doi: 10.2105/ajph.2018.304428
18. Boyd A, Golding J, Macleod J, et al. Cohort Profile: the 'children of the 90s'--the index offspring of the Avon Longitudinal Study of Parents and Children. *Int J Epidemiol* 2013;42(1):111-27. doi: 10.1093/ije/dys064
19. Fraser A, Macdonald-Wallis C, Tilling K, et al. Cohort Profile: the Avon Longitudinal Study of Parents and Children: ALSPAC mothers cohort. *Int J Epidemiol* 2013;42(1):97-110. doi: 10.1093/ije/dys066
20. Barter C, McCarry M, Berridge D, et al. Partner exploitation and violence in teenage intimate relationships. Bristol: NSPCC/University of Bristol 2009.
21. Hester M, Ferrari G, Jones SK, et al. Occurrence and impact of negative behaviour, including domestic violence and abuse, in men attending UK primary care health clinics: a cross-sectional survey. *BMJ Open* 2015;5(5):e007141. doi: 10.1136/bmjopen-2014-007141
22. Gadermann AM, Guhn M, Zumbo BD. Estimating ordinal reliability for Likert-type and ordinal item response data: A conceptual, empirical, and practical guide. *Practical Assessment, Research & Evaluation* 2012;17(3):1-13.
23. Holgado–Tello FP, Chacón–Moscoso S, Barbero–García I, et al. Polychoric versus Pearson correlations in exploratory and confirmatory factor analysis of ordinal variables. *Quality & Quantity* 2008;44(1):153-66. doi: 10.1007/s11135-008-9190-y
24. Yong AG, Pearce S. A beginner's guide to factor analysis: Focusing on exploratory factor analysis. *Tutorials in Quantitative Methods for Psychology* 2013;9(2):79-94.
25. Caldwell JE, Swan SC, Woodbrown VD. Gender differences in intimate partner violence outcomes. *Psychology of Violence* 2012;2(1):42-57. doi: 10.1037/a0026296

For peer review only

26. Johnson MP. Gender and types of intimate partner violence: A response to an anti-feminist literature review. *Aggression and Violent Behavior* 2011;16(4):289-96. doi: 10.1016/j.avb.2011.04.006
27. Eccles JS, Jacobs JE, Harold RD. Gender role stereotypes, expectancy effects, and parents' socialization of gender differences. *Journal of Social Issues* 1990;46(2):183-201.
28. WHO. Gender and mental health. Geneva: WHO 2002.
29. Jewkes R, Flood M, Lang J. From work with men and boys to changes of social norms and reduction of inequities in gender relations: a conceptual shift in prevention of violence against women and girls. *The Lancet* 2015;385(9977):1580-89. doi: 10.1016/s0140-6736(14)61683-4
30. Archer J. Sex differences in aggression between heterosexual partners: A meta-analytic review. *Psychological Bulletin* 2000;126(5):651-80. doi: 10.1037/0033-2909.126.5.651
31. DiStefano C, Zhu M, Mindrila D. Understand and using factor scores: Considerations for the applied researcher. *Practical Assessment, Research & Evaluation* 2009;14(20)
32. Campbell JC. Health consequences of intimate partner violence. *The Lancet* 2002;359(9314):1331-36. doi: 10.1016/s0140-6736(02)08336-8
33. Costello CG, Comrey AL. Scales for measuring depression and anxiety. *Journal of Psychology* 1967;66(2):303-14.

For peer review only

Appendix: PSYCHOMETRIC PROPERTIES AND GENDER DIFFERENCES IN IPV

Table A1: Polychoric correlations between ordinal IPV items

	Control	Humiliate	Push, slap	Punch, strangle	Coerced touch	Forced touch	Coerced sex	Forced sex
**Control**	1							
**Humiliate**	.764	1						
**Push, slap**	.728	.812	1					
**Punch, strangle**	.727	.786	.923	1				
**Coerced touch**	.592	.570	.630	.589	1			
**Forced touch**	.591	.569	.677	.649	.923	1		
**Coerced sex**	.627	.598	.640	.591	.882	.827	1	
**Forced sex**	.593	.602	.742	.720	.812	.890	.854	1

Note. N=3,158. Response categories for all variables were 0=never, 1=once, 2=a few times, and 3=often. Full item descriptions are shown in Table X.

Table A2: Exploratory factor analysis

	Single factor solution		Two-factor solution (r=64.97%)		
**Item**	**Factor 1**	**Uniqueness**	**Factor 1**	**Factor 2**	**Uniqueness**
**Control**	.771	.406	-	.705	.344
**Humiliate**	.792	.373	-	.842	.249
**Push, slap**	.881	.224	-	.877	.106
**Punch, strangle**	.855	.269	-	.905	.127
**Coerced touch**	.870	.243	.951	-	.098
**Forced touch**	.891	.207	.913	-	.092
**Coerced sex**	.866	.251	.862	-	.156
**Forced sex**	.898	.193	.770	-	.147
**Factor eigenvalue:**	**5.834**		**5.834**	**0.847**	
**Proportion of variance explained**	**.865**		**.746**	**.724**	

Note. N=3,158. Method is principal factors using a polychoric correlation matrix. Two-factor solution uses oblique rotation (promax). Full item descriptions are shown in Table 1.

Table A3: Polychoric correlations between IPV impact items

	Frightened	Upset	Affect work	Sad	Anxious	Depressed	Substance use	Angry	No effect	Loved	Funny
**Frightened**	1										
**Upset**	.717	1									
**Affect work**	.621	.721	1								
**Sad**	.663	.893	.799	1							
**Anxious**	.701	.693	.724	.631	1						
**Depressed**	.646	.732	.776	.778	.747	1					
**Substance use**	.379	.400	.480	.406	.452	.531	1				
**Angry**	.343	.660	.318	.558	.401	.426	.297	1			
**No effect**	-.627	-.862	-.590	-.820	-.686	-.678	-.264	-.631	1		
**Loved**	-.178	-.468	-.162	-.376	-.148	-.164	-.018	-.301	.419	1	
**Funny**	-.478	-.522	-.318	-.422	-.472	-.454	-.093	-.392	.639	.432	1

Note. N=1,092. Participants only responded if they had experienced any IPV victimisation. Response categories for all variables were 0=no, 1=yes. Full item descriptions are shown in Table 2.

For peer review only

Appendix: PSYCHOMETRIC PROPERTIES AND GENDER DIFFERENCES IN IPV

Table A4: Age of occurrence among those who had experienced IPV

		N (%)		
**Item**	**Total N**	**Under 18**	**Over 18**	**Both**
Told you who you could see and where you could go and/or regularly checked what you were doing and where you were (by phone or text)	953	201 (21.09)	569 (59.71)	183 (19.20)
Made fun of you, called you hurtful names, shouted at you	1,022	171 (16.73)	666 (65.17)	185 (18.10)
Used physical force such as pushing, slapping, hitting or holding you down	743	137 (18.44)	468 (62.99)	138 (18.57)
Used more severe physical force such as punching, strangling, beating you up, hitting you with an object	481	70 (14.55)	293 (60.91)	118 (24.53)
Pressured you into kissing/touching/something else	569	109 (19.16)	337 (59.23)	123 (21.62)
Physically forced you into kissing/touching/something else	437	74 (16.93)	255 (58.35)	108 (24.71)
Pressured you into having sexual intercourse	631	135 (21.39)	371 (58.80)	125 (19.91)
Physically forced you into having sexual intercourse	413	65 (15.74)	244 (59.08)	104 (25.18)

For peer review only

Table 1. **The STROBE Statement: a checklist of items that should be addressed in reports of observational studies**

Item		Item number	Recommendation
**Title and abstract**	pp. 1-2	1	(a) Indicate the study's design with a commonly used term in the title or the abstract<br>(b) Provide in the abstract an informative and balanced summary of what was done and what was found
**Introduction**			
**Background/rationale**	p. 3	2	Explain the scientific background and rationale for the investigation being reported
**Objectives**		3	State specific objectives, including any pre-specified hypotheses
**Methods**			
**Study design**	pp. 3-4	4	Present key elements of study design early in the paper
**Setting**	pp. 3-4	5	Describe the setting, locations and relevant dates, including periods of recruitment, exposure, follow-up and data collection
**Participants**	pp. 3-4	6	(a) *Cohort study* – Give the eligibility criteria, and the sources and methods of selection of participants. Describe methods of follow-up<br>*Case-control study* – Give the eligibility criteria, and the sources and methods of case ascertainment and control selection. Give the rationale for the choice of cases and controls<br>*Cross-sectional study* – Give the eligibility criteria, and the sources and methods of selection of participants<br>(b) *Cohort study* – For matched studies, give matching criteria and number of exposed and unexposed<br>*Case-control study* – For matched studies, give matching criteria and the number of controls per case
**Variables**	p. 4	7	Clearly define all outcomes, exposures, predictors, potential confounders and effect modifiers. Give diagnostic criteria, if applicable
**Data sources/measurement**	p. 4	8[a]	For each variable of interest, give sources of data and details of methods of assessment (measurement). Describe comparability of assessment methods if there is more than one group
**Bias**	p. 5	9	Describe any efforts to address potential sources of bias
**Study size**	pp. 3-4	10	Explain how the study size was arrived at
**Quantitative variables**	p. 5	11	Explain how quantitative variables were handled in the analyses. If applicable, describe which groupings were chosen and why
**Statistical methods**	p. 5	12	(a) Describe all statistical methods, including those used to control for confounding<br>(b) Describe any methods used to examine subgroups and interactions<br>(c) Explain how missing data were addressed<br>(d) *Cohort study* – If applicable, explain how loss to follow-up was addressed<br>*Case-control study* – If applicable, explain how matching of cases and controls was addressed<br>*Cross-sectional study* – If applicable, describe analytical methods taking account of sampling strategy<br>(e) Describe any sensitivity analyses
**Results**			
**Participants**	pp. 3-4	13[a]	(a) Report the numbers of individuals at each stage of the study – e.g. numbers potentially eligible, examined for eligibility, confirmed eligible, included in the study, completing follow-up and analyzed<br>(b) Give reasons for non-participation at each stage<br>(c) Consider use of a flow diagram
**Descriptive data**	pp. 5-6	14[a]	(a) Give characteristics of study participants (e.g. demographic, clinical, social) and information on exposures and potential confounders<br>(b) Indicate the number of participants with missing data for each variable of interest<br>(c) *Cohort study* – Summarize follow-up time (e.g. average and total amount)
**Outcome data**	pp. 6-8	15[a]	*Cohort study* – Report numbers of outcome events or summary measures over time<br>*Case-control study* – Report numbers in each exposure category, or summary measures of exposure<br>*Cross-sectional study* – Report numbers of outcome events or summary measures

For peer review only - http://bmjopen.bmj.com/site/about/guidelines.xhtml

(Table 1, cont.)

Item		Item number	Recommendation
**Main results**	pp. 6-8	16	(a) Give unadjusted estimates and, if applicable, confounder-adjusted estimates and their precision (e.g. 95% confidence interval). Make clear which confounders were adjusted for and why they were included<br>(b) Report category boundaries when continuous variables were categorized<br>(c) If relevant, consider translating estimates of relative risk into absolute risk for a meaningful time period
**Other analyses**	pp. 9-10	17	Report other analyses done – e.g. analyses of subgroups and interactions, and sensitivity analyses
**Discussion**			
**Key results**	pp. 11-12	18	Summarize key results with reference to study objectives
**Limitations**	p. 12	19	Discuss limitations of the study, taking into account sources of potential bias or imprecision. Discuss both direction and magnitude of any potential bias
**Interpretation**	pp. 11-12	20	Give a cautious overall interpretation of results considering objectives, limitations, multiplicity of analyses, results from similar studies, and other relevant evidence
**Generalizability**	p. 12	21	Discuss the generalizability (external validity) of the study results
**Other information**			
**Funding**	p. 12	22	Give the source of funding and the role of the funders for the present study and, if applicable, for the original study on which the present article is based

[a] Give such information separately for cases and controls in case-control studies, and, if applicable, for exposed and unexposed groups in cohort and cross-sectional studies.

An *Explanation and Elaboration* article[18–20] discusses each checklist item, and gives methodological background and published examples of transparent reporting. The STROBE checklist is best used in conjunction with this article (freely available on the web sites of *PLoS Medicine*, *Annals of Internal Medicine* and *Epidemiology*). Separate versions of the checklist for cohort, case-control and cross-sectional studies are available on the STROBE web site.

the checklist to other designs – e.g. case-crossover studies or ecological studies – and also to specific topic areas. Four extensions are now available for the CONSORT Statement.[21–24] A first extension to STROBE is under way for gene–disease association studies: the STROBE Extension to Genetic Association studies (STREGA) initiative.[25] We ask those who aim to develop extensions of the STROBE Statement to contact the coordinating group first to avoid duplication of effort.

The STROBE Statement should not be interpreted as an attempt to prescribe the reporting of observational research in a rigid format. The checklist items should be addressed in sufficient detail and with clarity somewhere in an article, but the order and format for presenting information depends on author preferences, journal style and the traditions of the research field. For instance, we discuss the reporting of results under several separate items, while recognizing that authors might address several items within a single section of text or in a table. Also, item 22, on the source of funding and the role of funders, could be addressed in an appendix or in the methods section of the article. We do not aim at standardizing reporting. Authors of randomized clinical trials were asked by an editor of a specialist medical journal to "CONSORT" their manuscripts on submission.[26] We believe that manuscripts should not be "STROBEd", in the sense of regulating style or terminology. We encourage authors to use narrative elements, including the description of illustrative cases, to complement the essential information about their study, and to make their articles an interesting read.[27]

We emphasize that the STROBE Statement was not developed as a tool for assessing the quality of published observational research. Such instruments have been developed by other groups and were the subject of a recent systematic review.[28] In the *Explanation and Elaboration* paper, we used several examples of good reporting from studies whose results were not confirmed in further research – the important feature was the good reporting, not whether the research was of good quality. However, if STROBE is adopted by authors and journals, issues such as confounding, bias and generalizability could become more transparent, which might help temper the over-enthusiastic reporting of new findings in the scientific community and popular media,[29] and improve the methodology of studies in the long term. Better reporting may also help to have more informed decisions about when new studies are needed and what they should address.

We did not undertake a comprehensive systematic review for each of the checklist items and sub-items, or do our own research to fill gaps in the evidence base. Further, although no one was excluded from the process, the composition of the group of contributors was influenced by existing networks and was not representative in terms of geography (it was dominated by contributors from Europe and North America) and probably was not representative in terms of research interests and disciplines. We stress that STROBE and other recommendations on the reporting of research should be seen as evolving documents that require continual assessment, refinement, and, if necessary, change. We welcome suggestions for the further dissemination of STROBE – e.g. by re-publication of the present article in specialist journals and in journals published in other languages. Groups or individuals who intend to translate the checklist to other languages should consult the coordinating group beforehand. We will revise the checklist in the future, taking into account comments, criticism, new evidence and experience

For peer review only - http://bmjopen.bmj.com/site/about/guidelines.xhtml

BMJ Open

Intimate partner violence victimisation in early adulthood: psychometric properties of a new measure and gender differences in the Avon Longitudinal Study of Parents and Children

Journal:	*BMJ Open*
Manuscript ID	bmjopen-2018-025621.R1
Article Type:	Research
Date Submitted by the Author:	27-Nov-2018
Complete List of Authors:	Yakubovich, Alexa; University of Oxford, Social Policy & Intervention<br>Heron, Jon; University of Bristol, School of Social and Community Medicine<br>Feder, Gene; University of Bristol, Community based medicine<br>Fraser, Abigail; University of Bristol, School of Social and Community Medicine<br>Humphreys, David; University of Oxford, Social Policy & Intervention
**Primary Subject Heading**:	Epidemiology
Secondary Subject Heading:	Public health, Mental health
Keywords:	SOCIAL MEDICINE, PUBLIC HEALTH, MENTAL HEALTH, EPIDEMIOLOGY

SCHOLARONE™
Manuscripts

Intimate partner violence victimisation in early adulthood: psychometric properties of a new measure and gender differences in the Avon Longitudinal Study of Parents and Children

Alexa R. Yakubovich,[1] Jon Heron,[2] Gene Feder,[2] Abigail Fraser,[2] David K. Humphreys[1,3]

[1]Department of Social Policy and Intervention, University of Oxford, Bristol, United Kingdom
[2]School of Social and Community Medicine, University of Bristol, Bristol, United Kingdom
[3]Green Templeton College, University of Oxford, Oxford, United Kingdom

Correspondence to: Ms. Alexa R. Yakubovich, Department of Social Policy and Intervention, University of Oxford, Oxford, OX1 2ER, United Kingdom, alexa.yakubovich@spi.ox.ac.uk

Word count (excluding title page, abstract, references, and tables): 3,000 words

For peer review only

ABSTRACT

Objectives: To evaluate the psychometric properties of a novel, brief measure of physical, psychological, and sexual intimate partner violence (IPV) and estimate the overall prevalence of and gender differences in this violence.

Design: Data are from the Avon Longitudinal Study of Parents and Children (ALSPAC), a birth-cohort study.

Setting: Avon, United Kingdom.

Participants: 2,126 women and 1,142 men who completed the questionnaire assessment at age 21.

Outcome measures: Participants responded to eight items on physical, psychological, and sexual IPV victimisation at age 21. Participants indicated whether the violence occurred before age 18 and/or after and led to any of eight negative impacts (e.g., fear). We estimated the prevalence of IPV and tested for gender differences using chi-squared or t-tests. We evaluated the IPV victimisation measure based on internal consistency (alpha coefficient), dimensionality (exploratory factor analysis), and convergent validity with negative impacts.

Results: Overall, 37% of participants reported experiencing any IPV and 28% experienced any IPV after age 18. Women experienced more frequent IPV, more acts of IPV, and more negative impacts than men ($p<.001$ for all comparisons). The IPV measure showed high internal consistency ($\alpha=.95$), strong evidence for unidimensionality, and was highly correlated with negative impacts ($r=.579$, $p<.001$).

Conclusions: The prevalence of IPV victimisation in the ALSPAC cohort was considerable for both women and men. The strong and consistent gender differences in the frequency and severity of IPV suggests clinically meaningful differences in experiences of this violence. The ALSPAC measure for IPV victimisation was valid and reliable, indicating its suitability for further aetiological investigations.

Keywords: Epidemiology; Mental health; Public health; Social medicine

Strengths and limitations of this study:

- This study is the first to evaluate a novel and relatively brief measure of physical, psychological, and sexual intimate partner violence using data from a long running, high quality birth-cohort study in the United Kingdom.
- Timing of violence was measured which allowed us to compute both the lifetime and early adulthood prevalence of intimate partner violence.
- We used a robust analysis strategy to test for gender differences in intimate partner violence, which included analyzing the impacts of this violence to determine the severity of clinical burdens among women and men.
- Details on specific incidents or perpetrators of intimate partner violence were not measured and the generalisability of study findings to the national population and other contexts should be investigated.

For peer review only

INTRODUCTION

Intimate partner violence (IPV) is the most common violence perpetrated against women worldwide with severe consequences, including mortality, injury, and mental health disorders.[1-3] The most recent estimates for the United Kingdom (UK) indicate that IPV, especially among women, should be a public health priority, with 23% of women and 11% of men reporting any physical, psychological, or sexual IPV in their lifetime.[4 5] However, designing interventions for IPV requires accurately measuring and understanding its burden. Unlike many public health problems, official (e.g., police or hospital-reported) data typically provide poor estimates since most people do not contact formal services after experiencing IPV.[6]

Although survey data on IPV tend to be viewed as more accurate, measurement quality varies widely. While single-term and vague items such as *violence* are insufficient to measure the complexity of IPV, multi-item scales vary in content and length. The most commonly used measure is currently the Conflict Tactics Scale,[7-9] which measures specific behaviours by a current or previous dating, cohabiting, or marital partner. However, the Conflict Tactics Scale has been criticised for measuring IPV only within the context of conflicts or disagreements and not measuring the intent (e.g., self-defense or harm) or impact of violence.[10 11] Other validated scales include the Composite Abuse Scale,[12] WHO multi-country survey,[13] Abusive Behavior Inventory,[14] Severity of Violence Against Women Scale,[15] Measure of Wife Abuse,[16] and the Extended-Hurt/Insult/Threaten/Scream tool.[17] However, several of these do not measure psychological IPV (including controlling behaviour)[13 15 17] and most are relatively long (>30 items),[12 14-16] risking response burden in larger or repeated-measures surveys.

In recent years, in response to the criticisms and limitations of existing measures, short-form measures of physical, psychological, and sexual IPV have been developed with emerging evidence of validity (e.g., among Canadian women: the short-form Composite Abuse Scale[18]). This study is the first psychometric evaluation of a short-form measure for physical, psychological, and sexual IPV developed in the UK, which uniquely also collected data on the impacts of this violence and sampled both women and men. We aimed to (1) evaluate the psychometric properties of this new instrument; (2) estimate the overall prevalence of IPV and its impacts; and (3) test for gender differences in a UK-based birth-cohort study. This is essential to developing aetiological evidence for IPV against women, which, as demonstrated by a recent systematic review of prospective-longitudinal studies, is severely limited outside the United States (US).[19]

METHOD

We used data from the Avon Longitudinal Study of Parents and Children (ALSPAC). The birth-cohort study has established trust among participants, who have been self-completing questionnaires since age 5 (now in early adulthood), using online questionnaires at later time points – both of which are ideal for measuring IPV. The sampling frame included all pregnant women resident in one of three health districts in Avon, UK due between 1 April 1991 and 31 December 1992.[20 21] The initial number of pregnancies enrolled was 14,541. When participating children were approximately age 7, eligible cases not in the study were contacted, increasing the sample to 15,427 pregnancies, with 14,775 live births (76% of eligible live births) – these children are our target sample. Ethical approval was obtained from the ALSPAC Ethics and Law Committee and Local Research Ethics Committees. A

For peer review only - http://bmjopen.bmj.com/site/about/guidelines.xhtml

searchable data dictionary is available at http://www.bristol.ac.uk/alspac/researchers/our-data/.

Measuring IPV

At age 21, 3,458 participants completed the online questionnaire, of whom 3,268 (2,126 women, 1,142 men) provided any data on IPV, making this our starting sample. The IPV measures described below (see Table 1) were based on a previous National Society for Prevention of Cruelty to Children (NSPCC) questionnaire used in a young population in Bristol,[22] with modified wording and additional items based on the PROVIDE questionnaire.[23] The development group consisted of IPV researchers (Christine Barter, Marianne Hester, Eszter Szilassy, and Gene Feder); the questionnaire was piloted for acceptability with the ALSPAC participant advisory group.

Main instrument: IPV victimisation

Eight items measured physical, psychological, and sexual IPV victimisation. A ninth item (feeling scared) was relevant to the impact of this violence and is therefore included with the impact items. Participants indicated the frequency of each item (0=never to 3=often) and whether the behaviour occurred before and/or after age 18, allowing for measurement of temporality.

Impacts of IPV

Ten items measured the psychosocial impacts of IPV. Eight items indicated negative impacts (e.g., upset). One item measured whether the violence had no effect and two measured positive effects (e.g., feeling loved).

Table 1: IPV victimisation and impact items

Order	Victimisation items: How often altogether have any of your partners ever done any of the following to you and how old were you?	Type of IPV
1	Told you who you could see and where you could go and/or regularly checked what you were doing and where you were (by phone or text)?	Psychological
2	Made fun of you, called your hurtful names, shouted at you?	Psychological
3	Used physical force such as pushing, slapping, hitting or holding you down?	Physical
4	Used more severe physical force such as punching, strangling, beating you up, hitting you with an object?	Physical
5	Pressured you into kissing/touching/something else?	Sexual/ psychological
6	Physically forced you into kissing/touching/something else?	Sexual
7	Pressured you into having sexual intercourse?	Sexual/ psychological
8	Physically forced you into having sexual intercourse?	Sexual
**Order**	**Impact items: How did you feel after they these things to you?**	**Dimension**
1	Did any of the above make you feel scared or frightened, or did any partner make you feel frightened in any other way?*	Negative
2	Upset/unhappy	Negative
3	Affected my work/studies	Negative
4	Made me feel sad	Negative
5	No effect/not bothered	Null
6	Anxious	Negative
7	Made me drink more alcohol/take more drugs	Negative
8	Felt loved/protected/wanted	Positive
9	Thought it was funny	Positive
10	Angry/annoyed	Negative
11	Depressed	Negative

Note. For each victimisation item, participants indicate the frequency of occurrence – where 0=never, 1=once, 2=a few times, 3=often – and age of occurrence, where 1=under 18, 2=over 18, 3=both. The question prompt included the following definition for partner: 'By partner we

For peer review only - http://bmjopen.bmj.com/site/about/guidelines.xhtml

mean anyone you have ever been out with or had a relationship with, long-term or short-term (including one night stands)'. For each impact item, participants indicated 'yes' or 'no' as to whether this is how the IPV they experienced affected them.
*This item was asked along with the victimisation items and was therefore measured on the 'frequency' response scale.

Analysis

For aim one, we evaluated the internal consistency, dimensionality, and convergent validity of the IPV victimisation scale. To determine internal consistency, we computed an alpha coefficient for the eight IPV victimisation items using the polychoric (rather than Pearson) correlation matrix, which accounts for variables being ordinal rather than continuous.[24] As the scale's dimensionality was unknown, we conducted an exploratory factor analysis using this matrix.[25] We decided the appropriate number of factors based on their eigenvalues (using Kaiser's criterion that >1 indicates a viable factor), scree plot, and theoretical plausibility.[26] If a two (or more) factor solution was favourable, we decided a priori to use oblique rotation since we expected differing dimensions of abuse to correlate. To test for possible gender differences in factor solutions, we also ran the exploratory factor analysis separately for women and men. To assess convergent validity, we computed the Pearson correlation between the average frequency of IPV experiences and sum total of negative impacts among those who had experienced any IPV. For this step, we first confirmed (via polychoric correlation) that the negative impacts of IPV were positively correlated with each other and negatively correlated with the positive and null impacts (Table 1).

For aim two, we computed the mean of participants' scores across the eight IPV items (reflecting the average frequency of IPV experiences, 0-3), the mean number of IPV acts experienced at least once (0-8), the mean number of negative impacts (0-8), the proportion of participants who experienced any IPV, and the prevalence of any IPV with at least one negative impact. For aim three, we tested for gender differences in each item and summary variable using chi-squared or two-sided t-tests, as appropriate. For the latter, when the Levene's test suggested that the variances of women's and men's scores were unequal, we computed a two-sided t-test for unequal variances.

Patient and public involvement

The IPV measure was based on the NSPCC questionnaire,[22] which was developed with a young person's advisory group, and the PROVIDE survey,[23] which was discussed with the PROVIDE patient and public involvement group. Additionally, ALSPAC has an advisory panel of >30 participants who meet bi-monthly to advise on study design, methodology, and acceptability. ALSPAC communicates with participants via regular newsletters and has an active website and social media presence.

RESULTS

Table 2 summarises sample characteristics by gender. Women and men were very similar on baseline socio-demographics: most were White and had characteristics of higher socioeconomic status. At age 21, most women and men saw themselves as completely heterosexual (83% women, 85% men), followed by a smaller proportion reporting at least some same-sex preferences (16% women, 13% men) and a small number indicating asexuality (<1%). More women (72%) than men (59%), however, had been in relationships longer than 3 months by age 18 and, by age 20, more women (12%) than men (6%) were living with partners or children.

For peer review only

Table 2: Socio-demographic characteristics of the sample by gender

	Women	Men
**Baseline**		
Ethnicity		
Non-White	134 (3.64)	138 (3.74)
White	3,545 (96.36)	3,552 (96.26)
At least one parent had higher than O-level education		
Yes	3,224 (55.29)	3,400 (54.76)
No	2,607 (44.71)	2,809 (45.24)
At least one parent part of lower social class (partly or unskilled occupation)		
Yes	1,150 (23.76)	1,167 (22.87)
No (Both parents in professional, managerial, or skilled occupations)	3,690 (76.24)	3,936 (77.13)
Mother married		
Yes	4,807 (75.30)	5,100 (74.53)
No	1,577 (24.70)	1,743 (25.47)
Lived with both biological parents		
Yes	4,489 (90.29)	4,830 (90.26)
No	483 (9.71)	521 (9.74)
**Early adulthood (Ages 18-21)**		
Longest relationship (at Age 18)		
More than 3 months	1,632 (72.18)	1,034 (58.78)
Less than or equal to 3 months	629 (27.82)	725 (42.22)
Living arrangements (at Age 20):		
One or both parents	1,200 (48.21)	819 (51.51)
Partner and/or children	307 (12.33)	98 (6.16)
Other	982 (39.45)	673 (42.33)
Sexual preference (at Age 21)		
Asexual	8 (0.37)	6 (0.51)
Any same-sex preferences	358 (16.63)	160 (13.72)
100% heterosexual	1,787 (83.00)	1,000 (85.76)

Reliability and validity

Correlations were strong between all IPV scale items, ranging from .57 (between experiencing humiliation/name-calling/shouting and forced sexual touch) to .92 (between forced and coerced touch) (Appendix, Table A1). The alpha coefficient was .95, indicating strong internal consistency.

The exploratory factor analysis suggested a one- or two-factor solution (see Appendix, Table A2 for factor loadings). Only the first factor had an eigenvalue more than 1 (5.834). All items loaded highly onto this factor (ranging from 0.771-0.898), which suggests that using a factor-based score for experiences of IPV overall would be a valid analytical method in this sample. The scree plot plateaued between the second and third factor, and as the second factor had an eigenvalue close to 1 (0.847), we also attempted a two-factor solution with oblique rotation. This two-factor solution fit the data well, indicating plausible dimensions for (a) physical and psychological IPV and (b) sexual IPV. This suggests that analyses using a latent variable approach could reliably analyse these two factors. The factor analysis did not support a three-factor solution: the third factor had a low eigenvalue (0.182) and no items with a loading greater than .30. Overall, results were similar when factor analyses were run separately by gender (Appendix, Tables A3-A4): all items loading highly onto a single factor and the same two-factor solution was identified for women and men.

As expected, the eight negative impacts were all positively correlated (ρ=.297-.893, Appendix, Table A5). These items were also negatively correlated with IPV having no impact, seeming funny, or increasing perceptions of being loved, protected, or wanted (ρ=-.264 to -.862). Finally, these three null or positive impacts were positively correlated (ρ=.419-.639). We therefore, as planned, correlated the sum total of the negative impacts of IPV with the average frequency of IPV experiences among those who had experienced any

For peer review only

IPV. As expected, experiencing more frequent IPV was strongly correlated with experiencing more negative impacts (N=1,111): r=.579, p<.001.

Overall prevalence

As shown in Table 3, the most frequently experienced IPV was psychological (e.g., 25% reported humiliation, name-calling, or shouting) and the least experienced was sexual (e.g., 4% reported forced sex). Among those who experienced any IPV, the majority of violent acts (>78%) occurred after age 18 (see Table A6, Appendix for more detail). Most participants reported at least one negative impact following IPV, with the most common being feeling upset (78%) or angry (75%). The least common impacts of IPV were the positive ones: 13% of participants reported that the violence made them feel loved, protected, or wanted; 14% found the violence amusing.

Overall, 37% of participants reported experiencing any IPV and 28% experienced any IPV after age 18. The mean number of IPV acts experienced among those who experienced any violence, ranging from 1 to 8, was 3.004 (SD=2.108) overall and 2.167 (SD=1.644) after age 18. The mean number of negative impacts, ranging from 0 to 8, was 3.950 (SD=2.371) among those who had experienced any IPV and 2.944 (SD=2.633) among those who had experienced IPV after age 18.

For peer review only

Table 3: Frequencies of 8 IPV victimisation and impact items

Victimisation items	Total N	N (%) Never	Once	A few times	Often
Told you who you could see and where you could go and/or regularly checked what you were doing and where you were (by phone or text)	3,268	2,544 (77.85)	124 (3.79)	322 (12.91)	178 (5.45)
Made fun of you, called you hurtful names, shouted at you	3,253	2,422 (74.45)	170 (5.23)	530 (16.29)	131 (4.03)
Used physical force such as pushing, slapping, hitting or holding you down	3,255	2,768 (85.04)	193 (5.93)	235 (7.22)	59 (1.81)
Used more severe physical force such as punching, strangling, beating you up, hitting you with an object	3,252	3,075 (94.56)	81 (2.49)	68 (2.09)	28 (0.86)
Pressured you into kissing/touching/something else	3,255	2,981 (96.58)	96 (2.95)	146 (4.49)	32 (0.98)
Physically forced you into kissing/touching/something else	3,250	3,115 (95.85)	68 (2.09)	49 (1.51)	18 (0.55)
Pressured you into having sexual intercourse	3,242	2,876 (88.71)	181 (5.58)	152 (4.69)	33 (1.02)
Physically forced you into having sexual intercourse	3,239	3,118 (96.26)	80 (2.47)	32 (0.99)	9 (0.28)
**Impact items**	**Total N**	**Never**	**Once**	**A few times**	**Often**
Scared or frightened in any way	3,221	2,711 (84.17)	191 (5.93)	234 (7.26)	85 (2.64)
**Impact items: Only those who experienced at least 1 act of IPV**	**Total N**	**Yes**		**No**	
Upset/unhappy	1,148	900 (78.40)		248 (21.60)	
Angry/annoyed	1,139	857 (75.24)		282 (24.76)	
Made me feel sad	1,142	813 (71.19)		329 (28.81)	
Affected my work/studies	1,141	799 (70.03)		342 (29.97)	
Anxious	1,133	495 (43.69)		638 (56.31)	
Depressed	1,138	418 (36.73)		720 (63.27)	
No effect/not bothered	1,133	206 (18.18)		927 (81.82)	
Made me drink more alcohol/take more drugs	1,138	168 (14.76)		970 (85.24)	
Thought it was funny	1,132	158 (13.96)		974 (86.04)	
Felt loved/protected/wanted	1,135	148 (13.04)		987 (86.96)	

For peer review only

Gender differences

As shown in Table 4, for all IPV victimisation items, regardless of whether lifetime or early adulthood (ages 18-21) was considered, significantly more women experienced violence than men. The largest percentage difference was for the lifetime prevalence of coerced sex (15% women, 4% men). Moreover, significantly more women than men reported experiencing all negative impacts of IPV, apart from substance use where there was no difference. The greatest percentage difference was in feeling scared because of their partner (56% women, 14% men in their lifetime). In contrast, more men than women reported that the IPV they experienced was funny or had no effect on them. Finally, every test indicated that women experienced more frequent and severe IPV overall than men, in both their lifetimes and early adulthood (Table 5): women experienced more frequent and more acts of IPV compared to men; more women than men experienced any IPV (with or without negative impact); and, among those who had experienced any IPV, women experienced more negative impacts than men.

For peer review only

Table 4: Gender differences in IPV victimisation and impact items

Victimisation items	Lifetime<br>Women (N=2,050)	Men (N=1,108)	χ^2 (p)	Ages 18-21<br>Women (N=2,014)	Men (N=1,092)	χ^2 (p)
Told you who you could see, where you could go, or regularly checked what you were doing and where you were	510 (24.88)	196 (17.69)	21.41 (<.001)	346 (17.18)	152 (13.92)	5.59 (.018)
Made fun of you, called you hurtful names, shouted at you	596 (29.07)	210 (18.95)	38.75 (<.001)	443 (22.00)	166 (15.20)	20.74 (<.001)
Used physical force such as pushing, slapping, hitting or holding you down	362 (17.66)	106 (9.57)	37.31 (<.001)	245 (12.16)	82 (7.51)	16.29 (<.001)
Used more severe physical force such as punching, strangling, beating you up, hitting you with an object	142 (6.93)	31 (2.80)	23.68 (<.001)	96 (4.77)	22 (2.01)	14.67 (<.001)
Pressured you into kissing/touching/something else	240 (11.71)	26 (2.35)	81.70 (<.001)	144 (7.15)	20 (1.83)	40.05 (<.001)
Physically forced you into kissing/touching/something else	125 (6.10)	7 (0.63)	53.65 (<.001)	72 (3.57)	5 (0.46)	28.46 (<.001)
Pressured you into having sexual intercourse	313 (15.27)	43 (3.88)	93.25 (<.001)	192 (9.53)	36 (3.30)	40.49 (<.001)
Physically forced you into having sexual intercourse	114 (5.56)	5 (0.45)	51.79 (<.001)	64 (3.18)	5 (0.46)	24.12 (<.001)
**Impact items (Among those who experienced any IPV)**	**Women (N=800)**	**Men (N=292)**	**χ^2 (p)**	**Women (N=552)**	**Men (N=221)**	**χ^2 (p)**
Scared	444 (55.50)	40 (13.70)	151.47 (<.001)	279 (50.54)	31 (14.03)	87.61 (<.001)
Upset/unhappy	684 (85.50)	179 (61.30)	75.58 (<.001)	465 (84.24)	141 (63.80)	38.92 (<.001)
Angry/annoyed	625 (78.12)	195 (66.78)	14.72 (<.001)	441 (79.89)	152 (68.78)	10.91 (.001)
Made me feel sad	621 (77.62)	157 (53.77)	59.44 (<.001)	425 (76.99)	122 (55.20)	36.22 (<.001)
Affected my work/studies	275 (34.38)	51 (17.47)	29.21 (<.001)	187 (33.88)	38 (17.19)	21.28 (<.001)
Anxious	406 (50.75)	73 (25.00)	57.60 (<.001)	272 (49.28)	60 (27.15)	31.53 (<.001)
Depressed	329 (41.12)	69 (23.63)	28.27 (<.001)	231 (41.85)	52 (23.53)	22.82 (<.001)
No effect/not bothered	109 (13.63)	89 (30.48)	40.94 (<.001)	74 (13.41)	59 (26.70)	19.57 (<.001)
Made me drink more alcohol/take more drugs	127 (15.88)	34 (11.64)	3.05 (.081)	92 (16.67)	30 (13.57)	1.14 (.287)
Thought it was funny	64 (8.00)	92 (31.51)	96.53 (<.001)	48 (8.70)	67 (30.32)	58.26 (<.001)
Felt loved/protected/wanted	101 (12.62)	41 (14.04)	0.38 (.538)	80 (14.49)	32 (14.48)	0.00 (.996)

Note. Victimisation items were coded as 1=experienced at least once, 0=never experienced. Impact items were 1=yes, 0=no.

Table 5: Summary statistics for comparisons between women and men on overall IPV victimisation and impact

Item	Lifetime<br>Women<br>N	M (SD) or N (%)	Men<br>N	M (SD) or N (%)	t(df) or χ^2	p	Ages 18-21<br>Women<br>N	M (SD) or N (%)	Men<br>N	M (SD) or N (%)	t(df) or χ^2	p
Mean frequency of IPV experiences (SD)	2,128	0.28 (0.50)	1,145	0.12 (0.25)	12.61 (3,252.18)	<.001	2,128	0.19 (0.39)	1,145	0.10 (0.24)	7.58 (3,219.11)	<.001
Mean number of IPV acts experienced (SD)	2,024	1.41 (2.19)	1,096	0.60 (1.22)	13.16 (3,115.22)	<.001	2,014	0.75 (1.47)	1,092	0.42 (0.97)	7.55 (2,996.44)	<.001
Any IPV (N, %)	2,024	851 (42.05)	1,096	318 (29.01)	51.53	<.001	2,014	612 (30.39)	1,092	250 (22.89)	19.83	<.001
Any IPV with a negative impact (N, %)	1,988	762 (38.33)	1,071	228 (21.29)	92.34	<.001	1,982	535 (26.99)	1,070	180 (16.82)	40.07	<.001
Mean number of negative impacts of IPV (SD)	800	4.39 (2.27)	292	2.73 (2.21)	10.75 (1,090)	<.001	746	3.21 (2.72)	279	2.24 (2.24)	5.77 (602.84)	<.001

Note. All t-tests were two-group t-tests with unequal variances, apart from 'number of negative impacts of IPV' for the overall sample, which did not have unequal variances between men and women (i.e., the Levene's test was statistically non-significant).

DISCUSSION

This study estimated the prevalence of physical, psychological, and sexual IPV in a UK birth cohort during early adulthood using a novel measure. The prevalence of IPV was high: 37% of participants had experienced any IPV in their lifetime and 28% had experienced IPV between ages 18 to 21. As in previous research, the most commonly experienced violence was psychological and the least commonly experienced was sexual.[5] Over three-quarters of those who had experienced IPV had experienced this violence when they were aged 18 or older. This aligns with the broader IPV literature, which has found that early adulthood is an especially high-risk period for experiencing IPV.[19] Most participants who had experienced IPV reported more than one negative psychological impact, with the most common being feeling upset or angry. The least common outcomes of IPV were finding the violence amusing or feeling more loved, wanted, or protected.

We found strong and consistent gender differences: for all types of violent behaviours, women experienced more frequent IPV than men, both in their lifetime and early adulthood. As in other prevalence surveys, the most dramatic differences between women and men were on sexual violence items.[5] For instance, the proportion of women who had ever experienced coerced sex was more than four times that of men. Moreover, significantly more women than men reported experiencing negative psychosocial impacts from IPV. For example, the proportion of women who felt afraid of their partner was more than four times that of men. Similar proportions of women and men reported that their alcohol and substance use increased after experiencing IPV. The evidence on whether there are gender differences in substance use following IPV is inconsistent;[27] one possible explanation for similar proportions is the greater psychological impacts of IPV among women balance with the greater baseline tendency among men to use substances.

In contrast, the proportion of men who found their experiences of IPV amusing was more than three times that of women. More than double the proportion of men also reported that this violence did not affect them. Together with the gender differences in the negative impacts of IPV, this suggests that women experience more severe IPV than men, which is more difficult to trivialise and more likely to cause psychological harm. This extends a large body of evidence demonstrating that women experience the majority of severe consequences of IPV and are more likely to have controlling, violent partners.[2 28] At the same time, participants' reporting on the impacts and interpretations of their IPV experiences may have been influenced by gender-role socialisation. Women may have more readily reported the negative consequences of violence from their partners whereas men may have felt more pressure to minimise their experiences and deny negative consequences due to internalised concepts of masculinity, for instance, as strong and powerful and femininity as vulnerable and weak.[29-31] Future survey research could explore this hypothesis by including questions on traditional gender-role attitudes.

Our findings contribute to the broader debate on gender asymmetry in IPV. Studies using the Conflict Tactics Scale or family conflict surveys tend to find equivalent prevalence estimates among women and men;[32] whereas crime or clinical surveys tend to find that women experience more IPV than men.[11 32] Our data came from a community-based birth-cohort study, using a measure without reference to crime or conflict resolution, which minimises these priming biases. Our findings demonstrate that women experience more frequent and severe IPV than men, but also confirm that a considerable number of men experience violence from their partners. Therefore, our results support the continued research and

For peer review only - http://bmjopen.bmj.com/site/about/guidelines.xhtml

advocacy enterprise for IPV against women in particular, while also demonstrating the need for resources to continue to be developed for both women and men.

The new measure for IPV tested in this study showed excellent internal consistency and a strong, positive correlation with negative impacts of IPV, indicating convergent validity. The exploratory factor analysis suggested that the measure could be reliably analysed as a single dimension of IPV or as two – (a) physical or psychological IPV and (b) sexual IPV. As the scale items all loaded highly onto a single factor, analysing a factor-based score would be appropriate and have the benefit of maintaining the measure's original scaling for more intuitive interpretation.[33] Overall, factor structures were equivalent among women and men. This should be confirmed in new samples, including tests of gender invariance, which overall has been understudied in the literature.[34]

Strengths and limitations

Study limitations include, firstly, not measuring the age of first occurrence of IPV before age 18. Second, the definition of intimate partner used was broad, from casual sex partners to long-term relationships. Since this could capture sexual violence by an acquaintance, future research should use a more constrained definition (e.g., dating or marital partner). Third, the ALSPAC instrument did not measure specific instances of IPV or specific relationships; it is therefore unclear whether IPV was experienced by multiple perpetrators or repeatedly during a single relationship. We are also unable to determine which types or instances of IPV caused the impacts reported. Although more time intensive, it would be useful if future uses of the ALSPAC instrument allowed participants to indicate the perpetrator(s) and impact(s) of each experience of IPV. Obtaining more detailed information on IPV events and the relationship context would help determine intent and precipitants to inform directions for intervention research. Relatedly, data from participants' partners or an equivalent measure of IPV perpetration were not collected in ALSPAC. However, in the absence of sampling partners, self-reported victimisation is a more sensitive measure of IPV than self-reported perpetration.[35 36] Moreover, although IPV experiences may have involved the use of violence as well, that a greater proportion of women experienced nearly every measured negative impact from IPV compared to men suggests important differences in the severity and experience of IPV that remain critical to consider both for research and clinical practice.

Fourth, the IPV impacts measured were mainly psychological, to the exclusion of further physical (e.g., injury) or socioeconomic consequences (apart from work or studies being affected). Additionally, the impact items were largely one-word terms such as *depressed* and *anxious*, which are more vulnerable to social desirability bias and internalised gender concepts as opposed to a scale of items measuring depression or anxiety.[37] Nevertheless, in longer surveys such as those used in ALSPAC, it may not be feasible to include more exhaustive measures of IPV impacts. Fifth, ALSPAC did not include alternative IPV measures to further evaluate the measure's convergent validity. Assessing convergence with long-form IPV measures in particular may be useful to determine if scale length or breadth has any impact on sensitivity or gender differences. Finally, higher socioeconomic positions and White persons are over-represented in this sample: the generalisability of our results to the greater UK population or other contexts requires further investigation.

Despite these limitations, our study has a number of strengths. The IPV measure used was brief and could therefore be implemented in surveys measuring a rich set of potential IPV predictors. Both women and men were sampled, allowing for analyses of gender differences.

For peer review only

Although age of first occurrence was not measured, participants indicated whether IPV occurred before and/or after age 18, which made it possible to compute a current prevalence of IPV (last three years) and allows for analyses of temporal relationships between antecedents and early adulthood IPV. Finally, our analyses were thorough and demonstrated consistent results, allowing for firm conclusions on the reliability of the IPV measure and gender differences in IPV in the ALSPAC cohort.

In conclusion, we found that more than one-third of participants engaged in a cohort study for over 20 years in the UK had experienced IPV by early adulthood. Women consistently experienced more frequent and severe IPV than men by all measures, suggesting important gender differences in the burden of this violence. The ALSPAC measure for IPV victimisation showed strong indicators of reliability and validity, demonstrating its appropriateness for further etiological studies.

Funding

The UK Medical Research Council and Wellcome (Grant ref: 102215/2/13/2) and the University of Bristol provide core support for ALSPAC. A comprehensive list of grants funding is available on the ALSPAC website (http://www.bristol.ac.uk/alspac/external/documents/grant-acknowledgements.pdf); this research was specifically funded by NHS Bristol Clinical Commissioning Group (RP-PG-0108-10048). ARY is funded by The Rhodes Trust and the Canadian Institutes of Health Research (DFS152265). Data access costs were additionally supported by the Department of Social Policy and Intervention Student Support Fund. AF and JH work at the MRC Integrative Epidemiology Unit which receives infrastructure funding from the UK Medical Research Council (MRC) (MC_UU_12013). AF is funded by a UK MRC fellowship awarded to AF (MR/M009351/1). GF is supported by the NIHR Biomedical Research Centre at University Hospitals Bristol NHS Foundation Trust and the University of Bristol.

Acknowledgments

The authors would like to thank all the families who took part in ALSPAC, the midwives who helped recruit them, and the whole ALSPAC team, including the interviewers, computer and laboratory technicians, clerical workers, research scientists, volunteers, managers, receptionists, and nurses.

Author contributions

ARY and DKH designed this study. ARY conducted the analyses, interpreted the data, and drafted the manuscript. GF, JH, AF, and DKH provided comments on the manuscript. All authors have read and approved this final version. This manuscript is the work of the authors, who will serve as guarantors for its contents. The views expressed in this manuscript are those of the author(s) and not necessarily those of the NHS, the National Institute for Health Research, or the Department of Health.

Competing interests None.

Data sharing statement Data underlying the results of this study are available from ALSPAC, based at the University of Bristol. Requests for data should be submitted to the ALSPAC Executive Committee at https://proposals.epi.bristol.ac.uk/ or sent to alspac-

For peer review only

data@bristol.ac.uk/. Details on all data are available through a fully searchable data dictionary at http://www.bristol.ac.uk/alspac/researchers/our-data/. Analysis syntax is stored by ARY and available upon request.

References

1. Krug EG, Dahlberg LL, Mercy JA, et al., editors. *World report on violence and health*. Geneva: World Health Organization, 2002.
2. Stöckl H, Devries K, Rotstein A, et al. The global prevalence of intimate partner homicide: a systematic review. *The Lancet* 2013;382(9895):859-65. doi: 10.1016/s0140-6736(13)61030-2
3. Coker AL, Davis KE, Arias I, et al. Physical and mental health effects of intimate partner violence for men and women. *Am J Prev Med* 2002;23(4):260-68. doi: 10.1016/s0749-3797(02)00514-7
4. Flatley J. Crime in England and Wales QMI. UK2018.
5. Flatley J. Domestic abuse findings from the Crime Survey for England and Wales year ending March 2017. UK: Office for National Statistics 2018.
6. Flatley J. Intimate personal violence and partner abuse. UK: Office for National Statistics 2016.
7. Straus MA. Measuring intrafamily conflict and violence: The conflict tactics (CT) scales. *Journal of Marriage and the Family* 1979:75-88.
8. Straus MA, Hamby SL, Boney-McCoy S, et al. The revised conflict tactics scales (CTS2) development and preliminary psychometric data. *Journal of family issues* 1996;17(3):283-316.
9. Straus MA, Douglas EM. A short form of the revised Conflict Tactics Scale. *Violence & Victims* 2004;19(5):520.
10. Walby S, Towers J, Balderston S, et al. The Concept and Measurement of Violence Against Women and Men. Bristol: Policy Press 2017.
11. Kimmel MS. "Gender symmetry" in domestic violence: A substantive and methodological research review. *Violence Against Women* 2002;8(11):1332-63. doi: 10.1177/107780102237407
12. Hegarty K, Bush R, Sheehan M. The Composite Abuse Scale: Further development and assessment of reliability and validity of a multidimensional partner abuse measure in clinical settings. *Violence and Victims* 2005;20(5):529-47.
13. García-Moreno C, Jansen HA, Ellsberg M, et al., editors. *WHO multi-country study on women's health and domestic violence against women: Initial results on prevalence, health outcomes and women's responses*. Geneva: World Health Organization, 2005.
14. Shepard MF, Campbell JA. The Abusive Behavior Inventory: A measure of psychological and physical abuse. *Journal of interpersonal violence* 1992;7(3):291-305.
15. Marshall LL. Development of the Severity of Violence Against Women Scales. *Journal of Family Violence* 1992;7(2):103-21.
16. Rodenburg FA, Fantuzzo JW. The Measure of Wife Abuse: Steps toward the development of a comprehensive assessment technique. *Journal of Family Violence* 1993;8(3):203-28.
17. Iverson KM, King MW, Gerber MR, et al. Accuracy of an intimate partner violence screening tool for female VHA patients: a replication and extension. *Journal of traumatic stress* 2015;28(1):79-82. doi: 10.1002/jts.21985

For peer review only

18. Ford-Gilboe M, Wathen CN, Varcoe C, et al. Development of a brief measure of intimate partner violence experiences: the Composite Abuse Scale (Revised)-Short Form (CASR-SF). *BMJ Open* 2016;6(12):e012824. doi: 10.1136/bmjopen-2016-012824
19. Yakubovich AR, Stöckl H, Murray J, et al. Risk and Protective Factors for Intimate Partner Violence Against Women: Systematic Review and Meta-analyses of Prospective–Longitudinal Studies. *American Journal of Public Health* 2018;108(7):e1-e11. doi: 10.2105/ajph.2018.304428
20. Boyd A, Golding J, Macleod J, et al. Cohort Profile: the 'children of the 90s'--the index offspring of the Avon Longitudinal Study of Parents and Children. *Int J Epidemiol* 2013;42(1):111-27. doi: 10.1093/ije/dys064
21. Fraser A, Macdonald-Wallis C, Tilling K, et al. Cohort Profile: the Avon Longitudinal Study of Parents and Children: ALSPAC mothers cohort. *Int J Epidemiol* 2013;42(1):97-110. doi: 10.1093/ije/dys066
22. Barter C, McCarry M, Berridge D, et al. Partner exploitation and violence in teenage intimate relationships. Bristol: NSPCC/University of Bristol 2009.
23. Hester M, Ferrari G, Jones SK, et al. Occurrence and impact of negative behaviour, including domestic violence and abuse, in men attending UK primary care health clinics: a cross-sectional survey. *BMJ Open* 2015;5(5):e007141. doi: 10.1136/bmjopen-2014-007141
24. Gadermann AM, Guhn M, Zumbo BD. Estimating ordinal reliability for Likert-type and ordinal item response data: A conceptual, empirical, and practical guide. *Practical Assessment, Research & Evaluation* 2012;17(3):1-13.
25. Holgado–Tello FP, Chacón–Moscoso S, Barbero–García I, et al. Polychoric versus Pearson correlations in exploratory and confirmatory factor analysis of ordinal variables. *Quality & Quantity* 2008;44(1):153-66. doi: 10.1007/s11135-008-9190-y
26. Yong AG, Pearce S. A beginner's guide to factor analysis: Focusing on exploratory factor analysis. *Tutorials in Quantitative Methods for Psychology* 2013;9(2):79-94.
27. Caldwell JE, Swan SC, Woodbrown VD. Gender differences in intimate partner violence outcomes. *Psychology of Violence* 2012;2(1):42-57. doi: 10.1037/a0026296
28. Johnson MP. Gender and types of intimate partner violence: A response to an anti-feminist literature review. *Aggression and Violent Behavior* 2011;16(4):289-96. doi: 10.1016/j.avb.2011.04.006
29. Eccles JS, Jacobs JE, Harold RD. Gender role stereotypes, expectancy effects, and parents' socialization of gender differences. *Journal of Social Issues* 1990;46(2):183-201.
30. WHO. Gender and mental health. Geneva: WHO 2002.
31. Jewkes R, Flood M, Lang J. From work with men and boys to changes of social norms and reduction of inequities in gender relations: a conceptual shift in prevention of violence against women and girls. *The Lancet* 2015;385(9977):1580-89. doi: 10.1016/s0140-6736(14)61683-4
32. Archer J. Sex differences in aggression between heterosexual partners: A meta-analytic review. *Psychological Bulletin* 2000;126(5):651-80. doi: 10.1037/0033-2909.126.5.651
33. DiStefano C, Zhu M, Mindrila D. Understand and using factor scores: Considerations for the applied researcher. *Practical Assessment, Research & Evaluation* 2009;14(20)
34. Caro BI. The revised Conflict Tactic Scales: one size does not fit all. Washington State University, 2017.
35. Archer J. Assessment of the reliability of the Conflict Tactic Scales: A meta-analytic review. *Journal of interpersonal violence* 1999;14(12):1263-89.

For peer review only

36. Cui M, Lorenz FO, Conger RD, et al. Observer, self-, and partner reports of hostile behaviors in romantic relationships. *Journal of Marriage and Family* 2005;67:1169-81.
37. Costello CG, Comrey AL. Scales for measuring depression and anxiety. *Journal of Psychology* 1967;66(2):303-14.

For peer review only

Appendix: PSYCHOMETRIC PROPERTIES AND GENDER DIFFERENCES IN IPV

Table A1: Polychoric correlations between ordinal IPV items

	Control	Humiliate	Push, slap	Punch, strangle	Coerced touch	Forced touch	Coerced sex	Forced sex
**Control**	1							
**Humiliate**	.764	1						
**Push, slap**	.728	.812	1					
**Punch, strangle**	.727	.786	.923	1				
**Coerced touch**	.592	.570	.630	.589	1			
**Forced touch**	.591	.569	.677	.649	.923	1		
**Coerced sex**	.627	.598	.640	.591	.882	.827	1	
**Forced sex**	.593	.602	.742	.720	.812	.890	.854	1

Note. N=3,158. Response categories for all variables were 0=never, 1=once, 2=a few times, and 3=often. Full item descriptions are shown in Table 1.

Table A2: Exploratory factor analysis (total sample)

	Single factor solution		Two-factor solution (r=64.97%)		
**Item**	**Factor 1**	**Uniqueness**	**Factor 1**	**Factor 2**	**Uniqueness**
**Control**	.771	.406	-	.705	.344
**Humiliate**	.792	.373	-	.842	.249
**Push, slap**	.881	.224	-	.877	.106
**Punch, strangle**	.855	.269	-	.905	.127
**Coerced touch**	.870	.243	.951	-	.098
**Forced touch**	.891	.207	.913	-	.092
**Coerced sex**	.866	.251	.862	-	.156
**Forced sex**	.898	.193	.770	-	.147
**Factor eigenvalue:**	**5.834**		**5.834**	**0.847**	
**Proportion of variance explained**	**.865**		**.865**	**.126**	

Note. N=3,158. Method is principal factors using a polychoric correlation matrix. Two-factor solution uses oblique rotation (promax). Factor loadings <.4 are suppressed. Full item descriptions are shown in Table 1.

Table A3: Exploratory factor analysis (women only)

	Single factor solution		Two-factor solution (r=63.86%)		
**Item**	**Factor 1**	**Uniqueness**	**Factor 1**	**Factor 2**	**Uniqueness**
**Control**	.786	.382	-	.714	.322
**Humiliate**	.805	.351	-	.863	.220
**Push, slap**	.886	.215	-	.887	.096
**Punch, strangle**	.860	.260	-	.928	.106
**Coerced touch**	.858	.264	.948	-	.107
**Forced touch**	.878	.230	.929	-	.093
**Coerced sex**	.860	.261	.853	-	.162
**Forced sex**	.892	.205	.755	-	.158
**Factor eigenvalue:**	**5.832**		**5.832**	**0.903**	
**Proportion of variance explained**	**.853**		**.853**	**.132**	

Note. N=2,050 women. Method is principal factors using a polychoric correlation matrix. Two-factor solution uses oblique rotation (promax). Factor loadings <.4 are suppressed. Full item descriptions are shown in Table 1.

For peer review only

Appendix: PSYCHOMETRIC PROPERTIES AND GENDER DIFFERENCES IN IPV

Table A4: Exploratory factor analysis (men only)

	Single factor solution		Two-factor solution (r=59.60%)		
**Item**	**Factor 1**	**Uniqueness**	**Factor 1**	**Factor 2**	**Uniqueness**
**Control**	.790	.377	-	.751	.294
**Humiliate**	.749	.439	-	.896	.248
**Push, slap**	.869	.245	-	.739	.188
**Punch, strangle**	.776	.398	-	.934	.189
**Coerced touch**	.876	.233	.928	-	.086
**Forced touch**	.926	.142	.646	-	.132
**Coerced sex**	.822	.325	.876	-	.192
**Forced sex**	.822	.325	.992	-	.104
**Factor eigenvalue:**	**5.518**		**5.518**	**1.049**	
**Proportion of variance explained**	**.690**		**.690**	**.131**	

Note. N=1,108 men. Method is principal factors using a polychoric correlation matrix, forced to be positive definite. Two-factor solution uses oblique rotation (promax). Factor loadings <.4 are suppressed. Full item descriptions are shown in Table 1.

Table A5: Polychoric correlations between IPV impact items

	Frightened	Upset	Affect work	Sad	Anxious	Depressed	Substance use	Angry	No effect	Loved	Funny
**Frightened**	1										
**Upset**	.717	1									
**Affect work**	.621	.721	1								
**Sad**	.663	.893	.799	1							
**Anxious**	.701	.693	.724	.631	1						
**Depressed**	.646	.732	.776	.778	.747	1					
**Substance use**	.379	.400	.480	.406	.452	.531	1				
**Angry**	.343	.660	.318	.558	.401	.426	.297	1			
**No effect**	-.627	-.862	-.590	-.820	-.686	-.678	-.264	-.631	1		
**Loved**	-.178	-.468	-.162	-.376	-.148	-.164	-.018	-.301	.419	1	
**Funny**	-.478	-.522	-.318	-.422	-.472	-.454	-.093	-.392	.639	.432	1

Note. N=1,092. Participants only responded if they had experienced any IPV victimisation. Response categories for all variables were 0=no, 1=yes. Full item descriptions are shown in Table 2.

Table A6: Age of occurrence among those who had experienced IPV

		N (%)		
**Item**	**Total N**	**Under 18**	**Over 18**	**Both**
Told you who you could see and where you could go and/or regularly checked what you were doing and where you were (by phone or text)	953	201 (21.09)	569 (59.71)	183 (19.20)
Made fun of you, called you hurtful names, shouted at you	1,022	171 (16.73)	666 (65.17)	185 (18.10)
Used physical force such as pushing, slapping, hitting or holding you down	743	137 (18.44)	468 (62.99)	138 (18.57)
Used more severe physical force such as punching, strangling, beating you up, hitting you with an object	481	70 (14.55)	293 (60.91)	118 (24.53)
Pressured you into kissing/touching/something else	569	109 (19.16)	337 (59.23)	123 (21.62)
Physically forced you into kissing/touching/something else	437	74 (16.93)	255 (58.35)	108 (24.71)
Pressured you into having sexual intercourse	631	135 (21.39)	371 (58.80)	125 (19.91)
Physically forced you into having sexual intercourse	413	65 (15.74)	244 (59.08)	104 (25.18)

For peer review only

Table 1. **The STROBE Statement: a checklist of items that should be addressed in reports of observational studies**

Item		Item number	Recommendation
**Title and abstract**	pp. 1-2	1	(a) Indicate the study's design with a commonly used term in the title or the abstract<br>(b) Provide in the abstract an informative and balanced summary of what was done and what was found
**Introduction**			
**Background/rationale**	p. 3	2	Explain the scientific background and rationale for the investigation being reported
**Objectives**		3	State specific objectives, including any pre-specified hypotheses
**Methods**			
**Study design**	pp. 3-4	4	Present key elements of study design early in the paper
**Setting**	pp. 3-4	5	Describe the setting, locations and relevant dates, including periods of recruitment, exposure, follow-up and data collection
**Participants**	pp. 3-4	6	(a) *Cohort study* – Give the eligibility criteria, and the sources and methods of selection of participants. Describe methods of follow-up<br>*Case-control study* – Give the eligibility criteria, and the sources and methods of case ascertainment and control selection. Give the rationale for the choice of cases and controls<br>*Cross-sectional study* – Give the eligibility criteria, and the sources and methods of selection of participants<br>(b) *Cohort study* – For matched studies, give matching criteria and number of exposed and unexposed<br>*Case-control study* – For matched studies, give matching criteria and the number of controls per case
**Variables**	p. 4	7	Clearly define all outcomes, exposures, predictors, potential confounders and effect modifiers. Give diagnostic criteria, if applicable
**Data sources/measurement**	p. 4	8[a]	For each variable of interest, give sources of data and details of methods of assessment (measurement). Describe comparability of assessment methods if there is more than one group
**Bias**	p. 5	9	Describe any efforts to address potential sources of bias
**Study size**	pp. 3-4	10	Explain how the study size was arrived at
**Quantitative variables**	p. 5	11	Explain how quantitative variables were handled in the analyses. If applicable, describe which groupings were chosen and why
**Statistical methods**	p. 5	12	(a) Describe all statistical methods, including those used to control for confounding<br>(b) Describe any methods used to examine subgroups and interactions<br>(c) Explain how missing data were addressed<br>(d) *Cohort study* – If applicable, explain how loss to follow-up was addressed<br>*Case-control study* – If applicable, explain how matching of cases and controls was addressed<br>*Cross-sectional study* – If applicable, describe analytical methods taking account of sampling strategy<br>(e) Describe any sensitivity analyses
**Results**			
**Participants**	pp. 3-4	13[a]	(a) Report the numbers of individuals at each stage of the study – e.g. numbers potentially eligible, examined for eligibility, confirmed eligible, included in the study, completing follow-up and analyzed<br>(b) Give reasons for non-participation at each stage<br>(c) Consider use of a flow diagram
**Descriptive data**	pp. 5-6	14[a]	(a) Give characteristics of study participants (e.g. demographic, clinical, social) and information on exposures and potential confounders<br>(b) Indicate the number of participants with missing data for each variable of interest<br>(c) *Cohort study* – Summarize follow-up time (e.g. average and total amount)
**Outcome data**	pp. 6-8	15[a]	*Cohort study* – Report numbers of outcome events or summary measures over time<br>*Case-control study* – Report numbers in each exposure category, or summary measures of exposure<br>*Cross-sectional study* – Report numbers of outcome events or summary measures

For peer review only - http://bmjopen.bmj.com/site/about/guidelines.xhtml

(Table 1, cont.)

Item		Item number	Recommendation
**Main results**	pp. 6-8	16	(a) Give unadjusted estimates and, if applicable, confounder-adjusted estimates and their precision (e.g. 95% confidence interval). Make clear which confounders were adjusted for and why they were included<br>(b) Report category boundaries when continuous variables were categorized<br>(c) If relevant, consider translating estimates of relative risk into absolute risk for a meaningful time period
**Other analyses**	pp. 9-10	17	Report other analyses done – e.g. analyses of subgroups and interactions, and sensitivity analyses
**Discussion**			
**Key results**	pp. 11-12	18	Summarize key results with reference to study objectives
**Limitations**	p. 12	19	Discuss limitations of the study, taking into account sources of potential bias or imprecision. Discuss both direction and magnitude of any potential bias
**Interpretation**	pp. 11-12	20	Give a cautious overall interpretation of results considering objectives, limitations, multiplicity of analyses, results from similar studies, and other relevant evidence
**Generalizability**	p. 12	21	Discuss the generalizability (external validity) of the study results
**Other information**			
**Funding**	p. 12	22	Give the source of funding and the role of the funders for the present study and, if applicable, for the original study on which the present article is based

[a] Give such information separately for cases and controls in case-control studies, and, if applicable, for exposed and unexposed groups in cohort and cross-sectional studies.

An *Explanation and Elaboration* article[18–20] discusses each checklist item, and gives methodological background and published examples of transparent reporting. The STROBE checklist is best used in conjunction with this article (freely available on the web sites of *PLoS Medicine*, *Annals of Internal Medicine* and *Epidemiology*). Separate versions of the checklist for cohort, case-control and cross-sectional studies are available on the STROBE web site.

the checklist to other designs – e.g. case-crossover studies or ecological studies – and also to specific topic areas. Four extensions are now available for the CONSORT Statement.[21–24] A first extension to STROBE is under way for gene–disease association studies: the STROBE Extension to Genetic Association studies (STREGA) initiative.[25] We ask those who aim to develop extensions of the STROBE Statement to contact the coordinating group first to avoid duplication of effort.

The STROBE Statement should not be interpreted as an attempt to prescribe the reporting of observational research in a rigid format. The checklist items should be addressed in sufficient detail and with clarity somewhere in an article, but the order and format for presenting information depends on author preferences, journal style and the traditions of the research field. For instance, we discuss the reporting of results under several separate items, while recognizing that authors might address several items within a single section of text or in a table. Also, item 22, on the source of funding and the role of funders, could be addressed in an appendix or in the methods section of the article. We do not aim at standardizing reporting. Authors of randomized clinical trials were asked by an editor of a specialist medical journal to "CONSORT" their manuscripts on submission.[26] We believe that manuscripts should not be "STROBEd", in the sense of regulating style or terminology. We encourage authors to use narrative elements, including the description of illustrative cases, to complement the essential information about their study, and to make their articles an interesting read.[27]

We emphasize that the STROBE Statement was not developed as a tool for assessing the quality of published observational research. Such instruments have been developed by other groups and were the subject of a recent systematic review.[28] In the *Explanation and Elaboration* paper, we used several examples of good reporting from studies whose results were not confirmed in further research – the important feature was the good reporting, not whether the research was of good quality. However, if STROBE is adopted by authors and journals, issues such as confounding, bias and generalizability could become more transparent, which might help temper the over-enthusiastic reporting of new findings in the scientific community and popular media,[29] and improve the methodology of studies in the long term. Better reporting may also help to have more informed decisions about when new studies are needed and what they should address.

We did not undertake a comprehensive systematic review for each of the checklist items and sub-items, or do our own research to fill gaps in the evidence base. Further, although no one was excluded from the process, the composition of the group of contributors was influenced by existing networks and was not representative in terms of geography (it was dominated by contributors from Europe and North America) and probably was not representative in terms of research interests and disciplines. We stress that STROBE and other recommendations on the reporting of research should be seen as evolving documents that require continual assessment, refinement, and, if necessary, change. We welcome suggestions for the further dissemination of STROBE – e.g. by re-publication of the present article in specialist journals and in journals published in other languages. Groups or individuals who intend to translate the checklist to other languages should consult the coordinating group beforehand. We will revise the checklist in the future, taking into account comments, criticism, new evidence and experience

For peer review only - http://bmjopen.bmj.com/site/about/guidelines.xhtml
